# A novel diagnostic model for tuberculous meningitis using Bayesian latent class analysis

**DOI:** 10.1186/s12879-024-08992-z

**Published:** 2024-02-06

**Authors:** Trinh Huu Khanh Dong, Joseph Donovan, Nghiem My Ngoc, Do Dang Anh Thu, Ho Dang Trung Nghia, Pham Kieu Nguyet Oanh, Nguyen Hoan Phu, Vu Thi Ty Hang, Nguyen Van Vinh Chau, Nguyen Thuy Thuong Thuong, Le Van Tan, Guy E. Thwaites, Ronald B. Geskus

**Affiliations:** 1 Centre for Tropical Medicine, Oxford University Clinical Research Unit, Ho Chi Minh City, Vietnam; 2 King’s College London, London, UK; 3 London School of Hygiene and Tropical Medicine, London, UK; 4 Centre for Tropical Medicine and Global Health, Nuffield Department of Medicine, University of Oxford, Oxford, UK; 5 the Hospital of Tropical Diseases, Ho Chi Minh City, Vietnam; 6 Pham Ngoc Thach University of Medicine, Ho Chi Minh City, Vietnam; 7 Ho Chi Minh City Department of Health, Ho Chi Minh City, Vietnam; 8 School of Medicine, Vietnam National University, Ho Chi Minh City, Vietnam

**Keywords:** Tuberculosis, Tuberculous meningitis, Diagnosis, Latent class model, Gold standard

## Abstract

**Background:**

Diagnosis of tuberculous meningitis (TBM) is hampered by the lack of a gold standard. Current microbiological tests lack sensitivity and clinical diagnostic approaches are subjective. We therefore built a diagnostic model that can be used before microbiological test results are known.

**Methods:**

We included 659 individuals aged $\ge 16$ years with suspected brain infections from a prospective observational study conducted in Vietnam. We fitted a logistic regression diagnostic model for TBM status, with unknown values estimated via a latent class model on three mycobacterial tests: Ziehl–Neelsen smear, Mycobacterial culture, and GeneXpert. We additionally re-evaluated mycobacterial test performance, estimated individual mycobacillary burden, and quantified the reduction in TBM risk after confirmatory tests were negative. We also fitted a simplified model and developed a scoring table for early screening. All models were compared and validated internally.

**Results:**

Participants with HIV, miliary TB, long symptom duration, and high cerebrospinal fluid (CSF) lymphocyte count were more likely to have TBM. HIV and higher CSF protein were associated with higher mycobacillary burden. In the simplified model, HIV infection, clinical symptoms with long duration, and clinical or radiological evidence of extra-neural TB were associated with TBM At the cutpoints based on Youden’s Index, the sensitivity and specificity in diagnosing TBM for our full and simplified models were 86.0% and 79.0%, and 88.0% and 75.0% respectively.

**Conclusion:**

Our diagnostic model shows reliable performance and can be developed as a decision assistant for clinicians to detect patients at high risk of TBM.

**Summary:**

Diagnosis of tuberculous meningitis is hampered by the lack of gold standard. We developed a diagnostic model using latent class analysis, combining confirmatory test results and risk factors. Models were accurate, well-calibrated, and can support both clinical practice and research.

**Supplementary Information:**

The online version contains supplementary material available at 10.1186/s12879-024-08992-z.

## Background

Tuberculous meningitis (TBM) is the most lethal form of *Mycobacterium tuberculosis* (*Mtb*) infection [1]. Early diagnosis is critical to initiate appropriate therapy, especially for low-middle-income countries, where access to diagnostic tests is limited. Nevertheless, diagnosing TBM is notoriously challenging as no gold standard exists. A confirmatory diagnosis of TBM requires identification of *Mtb* in cerebrospinal fluid (CSF), but conventional tests – Ziehl–Neelsen smear (ZN-Smear) microscopy, GeneXpert MTB/RIF (Xpert), and culture by Mycobacteria Growth Indicator Tube (MGIT) – lack sensitivity due to the challenge of Mtb identification in paucibacillary CSF [1, 2].

A diagnosis of TBM is often guided by a combination of clinical, biochemical, and imaging features. TBM is suggested by a long duration of symptoms at presentation and abnormal CSF biomarker values. A number of diagnostic algorithms have been created that utilise this information [3–6]. However, many of these have not been widely validated, especially for those individuals for whom the true disease status could not be verified.

In 2010, a uniform case definition (UCD) was developed based on expert opinion [5]. This illustrated the diagnostic challenges presented by TBM and was intended to facilitate comparison of research data, rather than for use in clinical practice. The UCD distinguishes between four diagnostic levels: *definite*, *probable*, *possible*, and *not* TBM. Individuals with microbiological or molecular confirmation of *Mtb* within the CSF are allocated to *definite* TBM. The remaining cases are defined as *probable* or *possible,* encompassing an expert-generated grading system where higher scores designate an increased probability of a diagnosis of TBM. It is expected that true TBM cases are represented by all *definite* cases, most *probable* cases, and some *possible* cases. Since TBM is almost always fatal if not treated with anti-tuberculosis drugs and delayed treatment is strongly associated with death or severe neurological sequelae in survivors, physicians usually err on the side of TBM treatment for those with compatible symptoms despite ongoing diagnostic uncertainty.

In social science and psychology, a technique named *Latent class analysis (LCA)* or *Latent class model (LCM)* has been employed that has the ability to infer “hidden groups” (called latent classes) based on “observed indicators”. The individual allocation into each group can be improved by relating it to “additional features”. LCA was later adopted in diagnostic studies to answer a similar problem when there is no practical gold standard to determine the disease status, as in the case of pulmonary tuberculosis and TBM [7–10].

The rationale and formulation of our LCA are discussed in the clinical and statistical supplementary appendices. In our study, we defined two “hidden groups”, those infected with Mtb and those infected with another pathogen, and the “observed indicators” are the three confirmatory tests (ZN-Smear, MGIT, and Xpert). The “additional features”, herein referred to as “diagnostic features” in our study, are the clinical, biochemical and imaging diagnostic features as used in the uniform case definition in combination with a few characteristics that are strongly indicative of not having TBM. A full list of included diagnostic features are outlined in the Clinical supplementary appendix. The main purpose of our study is to use the confirmatory test results and the diagnostic features to estimate TBM status per individual and develop a calibrated scoring system that quantifies the risk of TBM based on the diagnostic features. Our model allowed us to re-evaluate the performance of each confirmatory test. To improve clinical usability, we also developed a simplified scoring system requiring only clinical information and chest X-ray, without CSF analysis.

## Methods

### Participants

We used data from a prospective observational study on individuals with suspected brain infections who were admitted to the neuro-infection ward of the Hospital for Tropical Diseases (HTD), Ho Chi Minh City, Vietnam [11]. The HTD is a 550-bed centre providing secondary and tertiary treatment for a wide range of tropical infections in Southern Vietnam [3]. The study received ethical approval from HTD and the Oxford Tropical Research Ethics Committee and written informed consent was obtained from all participants or their relatives if they were incapacitated [11].

Participants were $\ge 16$ years old and were enrolled between 29th August 2017 and 22nd January 2021. All were suspected of neurological infection and underwent lumbar puncture at baseline as a routine diagnostic procedure. Patients were ineligible for enrolment if performing a lumbar puncture was contraindicated and excluded from our analysis if the mycobacterial cultures of CSF taken within the first week were contaminated.

### Data collection

#### Clinical and imaging data

Demographics (age, gender) and relevant medical history were collected. HIV tests were only conducted on those with identified risk factors for HIV infection. All participants underwent a chest X-ray. Brain imaging was not performed routinely and not included in the database.

#### Cerebrospinal fluid (CSF) analysis

White cell count (WCC) and cellular differential, protein, glucose (with paired blood glucose taken at the same time), and lactate were measured at enrolment. A Gram stain was performed to screen for bacterial meningitis, PCR/serology for viral meningitis, PCR for Angiostrongylus cantonensis, and India-ink staining and a cryptococcal antigen lateral flow test if cryptococcal meningitis was suspected. Where possible at least 6 mL CSF was used for mycobacterial testing by ZN-Smear, MGIT, and either Xpert MTB/RIF or Xpert MTB/RIF Ultra (Xpert Ultra). We combined Xpert Ultra with Xpert as they were diagnostically comparable for TBM in our setting [11]. In case an insufficient sample (< 5 mL) of CSF was collected, the microbiological tests were done according to case-by-case clinical judgement. In brief, if there was no clinical suggestion of TBM, the priority might be shifted to finding other diagnoses. If CSF was repeatedly sampled (as directed by clinical need), only the first sample was used as long as it had at least 3 mL of CSF and was not collected later than 7 days since enrolment. Methods of CSF processing have been described elsewhere [11].

#### Diagnosis and treatment

All patients received treatment according to the national and local guidelines. At the time of discharge or death, all were given a final diagnosis, based on the available clinical and laboratory information, including treatment response. If at least one of ZN-Smear, Xpert, or MGIT from CSF was positive at any time, the patient had *definite TBM*. Patients had *suspected TBM* if confirmatory microbiological and molecular tests were negative, but TBM was clinically suggested and anti-TB drugs were started. Those who recovered without anti-TB chemotherapy or had an alternative diagnosis confirmed microbiologically (e.g., by culture, PCR, or antigen tests) were assigned another diagnosis (i.e., *not TBM*).

### Statistical analysis

**Fig. 1:**
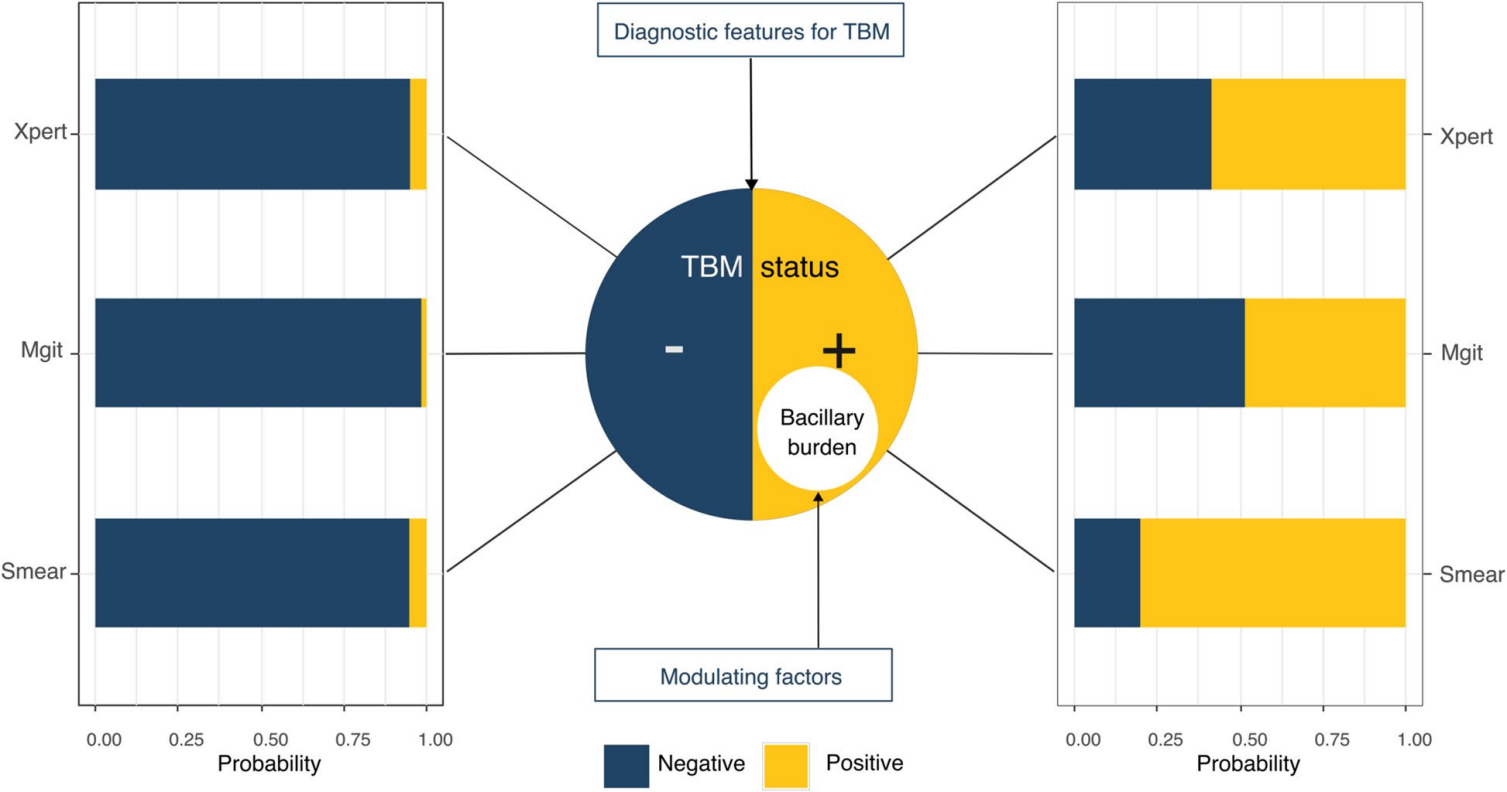
Basic model design. Unknown TBM status is linked with test results. The probability of a positive test depends on bacillary burden, which in turn depends on modulating factors. TBM risk factors help determining an individual’s TBM status. The distribution of test results shown on the bar plots are for demonstration only and do not correspond to the actual numbers

Our latent class model for TBM has two components, both consisting of logistic regression models (Fig. 1). In the *indicator model,* we made the results of three observed confirmatory tests ZN-Smear, MGIT, and Xpert depending on TBM status. The *prevalence model* quantifies the probability to have TBM. This probability is purely based on the diagnostic features, prior to any confirmatory tests. As the three tests share similar mechanisms of detecting the presence of *Mtb*, we added an individual random effect – which we call the mycobacillary burden – to eliminate their collinearity [7, 12]. The latent mycobacillary burden was related to a set of modulating factors (*bacillary burden sub-model*).

The choice of diagnostic features and modulating factors was based on prior knowledge of their association with TBM status and bacterial burden [5] (Clinical supplementary appendix). We added three indicators of an alternative diagnosis to the prevalence model: (1) positive CSF eosinophil count—a strong biomarker for eosinophilic meningitis, a relatively common condition in Vietnam, usually caused by *Angiostrongylus cantonensis*; (2) positive CSF Indian-ink staining or cryptococcal antigen lateral flow test; and (3) positive CSF Gram stain for non-acid-fast bacterial meningitis. We also included CSF red cell count as it is a marker of a traumatic lumbar puncture, which requires corrections to WCC and biochemical features [13–15]. A complete formulation of our model design is reported in the Statistical supplementary appendix.

We used the individual estimated TBM risk from the above model to fit a *simplified prevalence model* that excluded laboratory information. In their stead, we added some simple clinical symptoms involved in a TBM diagnosis: fever, headache, neck stiffness, and psychosis. These variables were easy to measure and less prone to missingness. This allowed us to create a simple risk score table for quick screening. Furthermore, we used Bayes’ rule to calculate the change in probability of having TBM after retrieving results of some or all confirmatory tests, relative to the pre-test TBM probability (Clinical supplementary appendix). Because any positive test means definite TBM, and because MGIT culture results take 2–8 weeks, we limited to five scenarios where negative confirmatory test results become available: *(a)* only Smear, *(b)* only Xpert, *(c)* Smear and MGIT, *(d)* Smear and Xpert, and *(e)* all three tests.

Missing values were handled based on their expected missingness mechanism (Clinical supplementary appendix). We later tested their validity by conducting appropriate sensitivity analyses in the Statistical supplementary appendix.

We chose a Bayesian approach for estimation and incorporated prior knowledge [1, 16, 17] on test sensitivity and specificity. on test sensitivity and specificity. Weakly informative prior distributions were used for all model coefficients (Statistical supplementary appendix). Estimates of the posterior distributions were obtained via Hamiltonian Markov Chain Monte Carlo using R version 4.2 [18] and Stan 2.27 [19]. Convergence was evaluated by the Brooks-Gelman-Rubin $\widehat{R}$ statistic. All results are presented as medians and equal-tailed 95% credible intervals (CrI) unless specified otherwise.

We compared different models and selected the best one using the expected log point-wise predictive density (elpd) [20]. To give an informal insight, we validated our full and simplified prevalence model with the final hospital diagnosis as a pseudo gold standard—in which a patient is considered as having TBM if they were diagnosed with suspected or definite TBM. A proper validation of the selected model was performed on the observed confirmatory test results and is explained in the Statistical supplementary appendix, in which we also compared our estimated TBM probability with standard UCD [5]. All performance metrics were calculated using repeated cross-validation procedures.

## Results

### Clinical characteristics

**Fig. 2:**
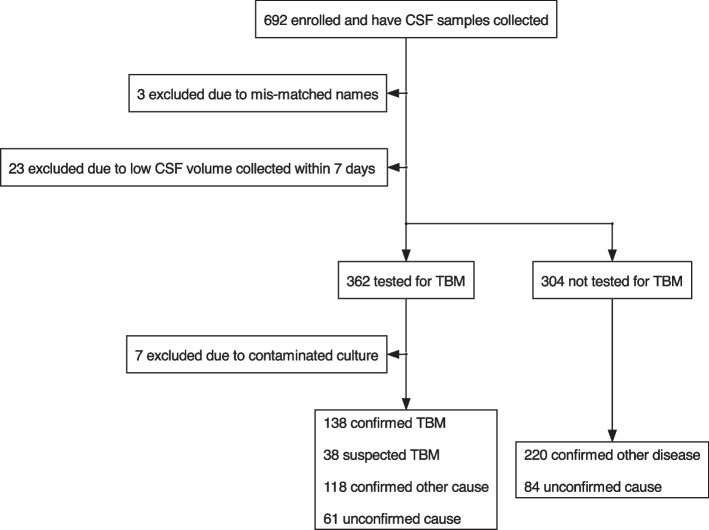
Participant recruitment flow

**Fig. 3:**
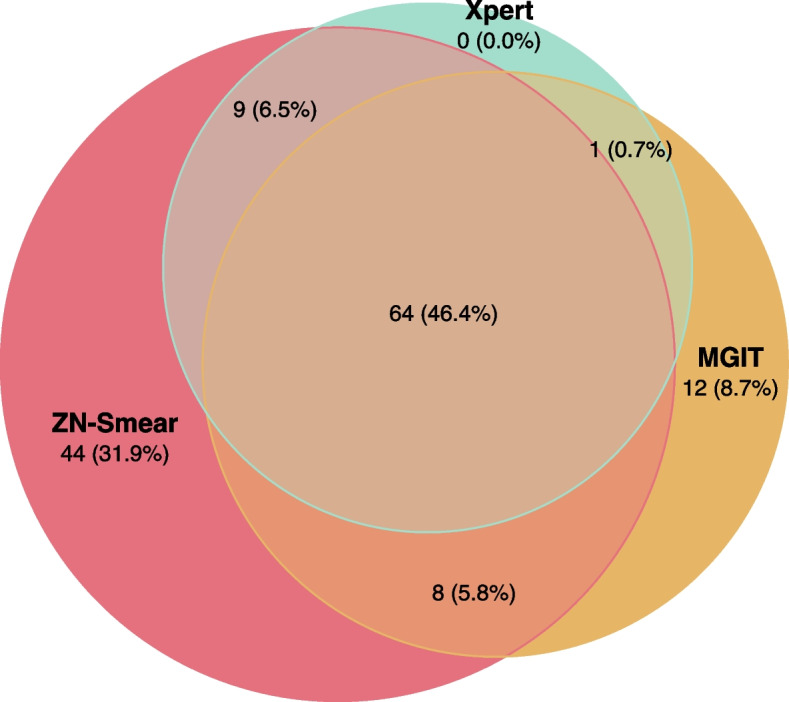
Venn diagram for ZN-Smear, MGIT, and Xpert profile in the study population

Of the 692 participants, 659 were included in the analysis (Fig. 2). All participants had at least one symptom suggestive of a brain infection (headache, fever, neck stiffness, vomiting, convulsion). Characteristics of included individuals are summarised in Table 1, stratified by overall and specific confirmatory test results. The median age of included individuals was 40 years. HIV co-infection status was assessed for 448/659 (68%) individuals, amongst whom 50 (11%) were positive. For each of the confirmatory tests, both HIV prevalence and duration from symptom onset to hospitalisation were higher in the test-positive group than in the negative and missing groups. There were 355 participants with valid TBM confirmatory test results, of whom 138 were microbiologically confirmed TBM (Fig. 3), while 118 were later confirmed with another disease. Amongst the 304 patients who were not tested microbiologically for TBM, 220 were confirmed with another pathogen. For 145 patients, from both the tested and the untested groups, no confirmed diagnosis could be made.

**Table 1:** Baseline characteristics of all study participants

	**Any test**				**ZN-Smear**		**MGIT**		**Xpert**	
**Characteristic**	***N* = 659**	**Positive, *N* = 138**^**a**^	**Negative, *N* = 217**^**a**^	**Missing, *N* = 304**^**a**^	**Positive, *N* = 125**^**a**^	**Negative, *N* = 230**^**a**^	**Positive, *N* = 85**^**a**^	**Negative, *N* = 270**^**a**^	**Positive, *N* = 74**^**a**^	**Negative, *N* = 281**^**a**^
Age (year(s))	659	42; 39 (29, 51)	42; 40 (27, 56)	42; 40 (26, 56)	42; 39 (29, 51)	42; 40 (27, 56)	40; 39 (29, 48)	43; 40 (28, 56)	40; 37 (29, 48)	43; 40 (28, 56)
HIV positive	448	33 (27%)	8 (5.2%)	9 (5.3%)	31 (27%)	10 (6.1%)	26 (33%)	15 (7.5%)	26 (37%)	15 (7.2%)
- *Missing*		14	63	134	11	66	6	71	4	73
Symptom duration (day(s))	633	15; 12 (8, 16)	12; 8 (5, 14)	9; 5 (3, 8)	15; 12 (8, 15)	12; 8 (5, 14)	18; 14 (10, 20)	12; 8 (5, 14)	18; 14 (10, 21)	12; 8 (5, 14)
- *Missing*		8	9	9	7	10	7	10	7	10
TB-suggested systemic symptoms	394	53 (58%)	42 (31%)	20 (12%)	49 (60%)	46 (32%)	36 (68%)	59 (34%)	33 (70%)	62 (35%)
- *Missing*		47	83	135	44	86	32	98	27	103
Noticed TB contact within the last 12 months	659	7 (5.1%)	3 (1.4%)	0 (0%)	7 (5.6%)	3 (1.3%)	4 (4.7%)	6 (2.2%)	4 (5.4%)	6 (2.1%)
Focal neurological deficit	643	24 (18%)	27 (13%)	28 (9.4%)	23 (19%)	28 (13%)	17 (20%)	34 (13%)	16 (22%)	35 (13%)
- *Missing*		4	7	5	4	7	2	9	2	9
Cranial nerve palsy	659	31 (22%)	27 (12%)	24 (7.9%)	29 (23%)	29 (13%)	25 (29%)	33 (12%)	23 (31%)	35 (12%)
Glasgow Coma Score	638	12; 12 (10, 15)	12; 12 (10, 14)	12; 12 (10, 13)	12; 12 (10, 15)	12; 12 (10, 14)	11; 11 (10, 14)	12; 13 (10, 15)	11; 11 (9, 14)	12; 13 (10, 15)
- *Missing*		1	4	16	1	4	1	4	1	4
X-ray pulmonary TB (excluding miliary TB)	659	22 (16%)	5 (2.3%)	4 (1.3%)	20 (16%)	7 (3.0%)	17 (20%)	10 (3.7%)	18 (24%)	9 (3.2%)
X-ray miliary TB	659	4 (2.9%)	0 (0%)	1 (0.3%)	4 (3.2%)	0 (0%)	4 (4.7%)	0 (0%)	3 (4.1%)	1 (0.4%)
CSF lymphocyte count (cell(s)/mm^3^)	656	228; 153 (82, 302)	278; 80 (13, 291)	270; 48 (5, 261)	220; 152 (81, 292)	279; 90 (14, 293)	237; 152 (81, 329)	265; 102 (21, 290)	212; 123 (57, 285)	271; 110 (23, 293)
- *Missing*		0	0	3	0	0	0	0	0	0
CSF WCC (cell(s)/mm^3^)	656	481; 300 (163, 559)	708; 140 (18, 460)	1,940; 68 (8, 856)	483; 280 (160, 564)	693; 164 (24, 478)	492; 330 (190, 519)	659; 166 (32, 479)	485; 300 (184, 614)	655; 176 (35, 475)
- *Missing*		0	0	3	0	0	0	0	0	0
CSF eosinophil count (cell(s)/mm^3^)	658	0; 0 (0, 0)	14; 0 (0, 0)	5; 0 (0, 0)	0; 0 (0, 0)	13; 0 (0, 0)	0; 0 (0, 0)	11; 0 (0, 0)	0; 0 (0, 0)	11; 0 (0, 0)
- *Missing*		0	0	1	0	0	0	0	0	0
CSF RBC Count (cell(s)/mm^3^)	659	874; 25 (2, 123)	2,040; 12 (0, 324)	1,673; 18 (0, 1,000)	341; 22 (2, 90)	2,263; 14 (0, 390)	1,164; 25 (0, 140)	1,719; 14 (0, 300)	267; 21 (0, 105)	1,934; 16 (0, 300)
CSF protein (g/l)	657	2.44; 1.79 (1.18, 2.45)	1.48; 0.88 (0.46, 1.75)	1.73; 0.73 (0.37, 1.98)	2.49; 1.86 (1.21, 2.46)	1.50; 0.91 (0.46, 1.82)	2.26; 1.89 (1.35, 2.50)	1.72; 1.01 (0.50, 1.93)	2.85; 1.88 (1.51, 2.39)	1.59; 1.00 (0.51, 1.97)
- *Missing*		0	0	2	0	0	0	0	0	0
CSF lactate (mmol/l)	657	5.8; 5.4 (3.8, 7.8)	4.1; 3.1 (2.3, 4.6)	5.6; 3.0 (2.2, 6.8)	5.9; 5.4 (3.8, 7.8)	4.2; 3.2 (2.3, 5.0)	6.5; 6.1 (4.4, 8.0)	4.3; 3.4 (2.3, 5.0)	6.8; 6.6 (5.2, 8.3)	4.3; 3.4 (2.3, 5.0)
- *Missing*		0	0	2	0	0	0	0	0	0
CSF glucose (mmol/l)	657	2.21; 1.97 (1.34, 2.80)	3.50; 3.51 (2.64, 4.31)	3.55; 3.76 (2.67, 4.54)	2.14; 1.97 (1.30, 2.76)	3.47; 3.49 (2.59, 4.30)	2.00; 1.76 (1.27, 2.29)	3.32; 3.33 (2.47, 4.08)	1.79; 1.67 (1.19, 2.18)	3.32; 3.31 (2.42, 4.08)
- *Missing*		0	0	2	0	0	0	0	0	0
Paired blood glucose (mmol/l)	651	6.55; 6.41 (5.80, 7.26)	6.96; 6.53 (5.44, 7.51)	7.17; 6.54 (5.45, 8.13)	6.51; 6.40 (5.78, 7.20)	6.96; 6.53 (5.46, 7.52)	6.60; 6.53 (5.78, 7.28)	6.87; 6.48 (5.52, 7.46)	6.49; 6.40 (5.70, 7.14)	6.89; 6.49 (5.53, 7.50)
- *Missing*		1	2	5	1	2	1	2	1	2
Cryptococcal antigen/Indian ink +	572	1 (0.7%)	5 (2.4%)	13 (5.8%)	1 (0.8%)	5 (2.3%)	1 (1.2%)	5 (1.9%)	1 (1.4%)	5 (1.8%)
- *Missing*		1	8	78	1	8	0	9	0	9
Positive CSF Gram stain	644	0 (0%)	7 (3.2%)	37 (13%)	0 (0%)	7 (3.1%)	0 (0%)	7 (2.6%)	0 (0%)	7 (2.5%)
- *Missing*		5	1	9	3	3	3	3	1	5
Fever	659	134 (97%)	203 (94%)	276 (91%)	121 (97%)	216 (94%)	82 (96%)	255 (94%)	71 (96%)	266 (95%)
Headache	606	127 (96%)	162 (81%)	215 (79%)	115 (96%)	174 (82%)	79 (98%)	210 (83%)	68 (96%)	221 (84%)
- *Missing*		6	16	31	5	17	4	18	3	19
Neck stiffness	633	75 (56%)	114 (54%)	158 (55%)	67 (56%)	122 (54%)	46 (55%)	143 (54%)	39 (54%)	150 (55%)
- *Missing*		5	4	17	5	4	2	7	2	7
Psychosis	647	4 (2.9%)	32 (15%)	63 (21%)	3 (2.4%)	33 (15%)	3 (3.6%)	33 (12%)	2 (2.7%)	34 (12%)
- *Missing*		2	3	7	2	3	1	4	1	4

In the “Any test” stratum: positive where at least one amongst ZN-Smear, MGIT, or Xpert is positive. ZN-Smear, MGIT, and Xpert are the sub-populations where the respective microbacterial test was performed (*N* = 355)

^a^Mean; Median (1^st^, 3^rd^ quartiles) for numeric variables; n (%) for categorical variables

### Model estimates

**Fig. 4:**
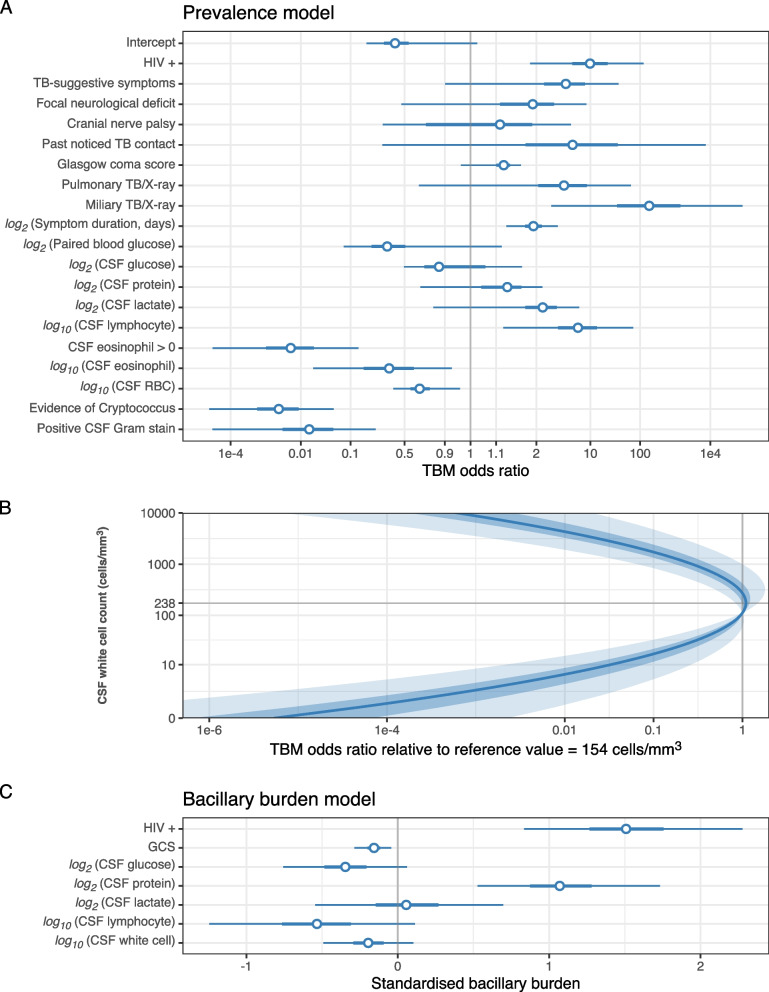
Posterior estimates of our selected model. **A** Prevalence model: TBM odds ratios by diagnostic features, except WCC; **B** TBM odds ratio by CSF WCC ($cells/m{m}^{3}$) over reference value = 154 $cells/m{m}^{3}$; **C** Modulating factors impacting individual bacillary burden, given they have TBM. In **A** and **C**: dot, thick and thin lines are medians, 50% and 95% credible intervals. In **B**: the blue line is median odds ratio, the inner and outer ribbon are 50% and 95% credible intervals

Estimated coefficients for TBM diagnostic features in the prevalence model are shown in Fig. 4 and the Clinical supplementary appendix. HIV infection, miliary TB, and longer symptom duration were associated with a higher risk of TBM (median OR = 9.9, 166.2, and 1.9, respectively). Of the laboratory variables, there was a strong increase in TBM risk with higher lymphocyte count (OR = 6.4, 95% CrI 1.2 – 69.1) and weaker increase with lower paired blood glucose (OR = 0.3, 0.1 – 1.2), lower CSF glucose (OR = 0.9, 0.5 – 1.5), higher CSF protein (OR = 1.2, 0.7 – 2.3), and higher lactate (OR = 2.3, 0.8 – 6.6). The relationship between CSF WCC and TBM risk was nonlinear, peaking at 238 cells per mm^3^ of CSF. HIV infection and higher CSF protein were associated with higher mycobacillary burden, while higher Glasgow coma score (GCS), CSF glucose, WCC and lymphocyte count all associated with lower mycobacillary burden.

Estimates of sensitivity and specificity of ZN-Smear, MGIT, and Xpert in diagnosing TBM are reported in Table 2. The specificity of each is > 99%. ZN-Smear was the most sensitive test with sensitivity of 62.5% (47.8%—80.9%) for people without HIV and 89.0% (81.5%—94.6%) for those with HIV. Xpert was the least sensitive, especially in the former group, at 33.8% (24.9%—45.7%). The sensitivity of Xpert was much higher in the group with HIV, at 75.5% (64.7%—84.3%) and comparable to MGIT sensitivity.

**Table 2:** Posterior estimates of test specificities and sensitivities to diagnose TBM, for overall population, and stratified by HIV infection. Test specificities are the same for all strata

	**Specificity (%)**	**Sensitivity (%)**		
**Test**	**(All strata)**	**Overall**	**HIV infected**	**HIV naive**
ZN-Smear	99.9 (99.2—100.0)	64.6 (50.9—81.8)	89.0 (81.5—94.6)	62.5 (47.8—80.9)
MGIT	99.7 (98.4—100.0)	42.1 (33.0—52.7)	75.9 (65.7—84.2)	39.1 (29.6—50.4)
Xpert	99.9 (99.4—100.0)	37.2 (28.7—48.3)	75.5 (64.7—84.3)	33.8 (24.9—45.7)

Values are in the form Median (95% Credible interval)

### Model pseudo-performance on diagnosing TBM

**Fig. 5:**
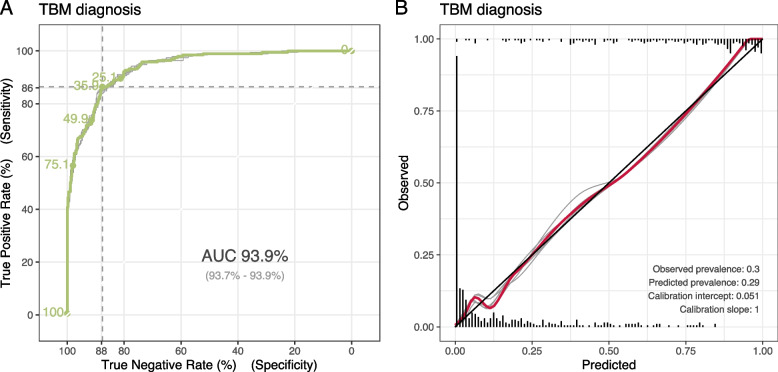
Performance of the selected prevalence model, assuming the final hospital diagnosis is the true status. **A** ROC curve and AUC: AUC values are presented as “average (min—max over 5 repetitions of cross-validation)”; **B** Calibration plot, showing the relationship between the predicted probability and observe outcome, smoothed by a loess curve. The grey lines are fitted curves from each 20-fold cross validation and coloured lines represents their average. The cross-validation procedure is explained in the Statistical supplementary appendix

Our selected indicator model showed good discrimination with the Areas under the Receiver-operating-characteristic curve (AUCs) of 92.6% for ZN Smear, 93.0% for MGIT, and 96.1% for Xpert (Statistical supplementary appendix). Assuming that the final hospital diagnosis was perfect, our selected prevalence model showed good discrimination with AUC = 93.9% (Fig. 5A). At the cutpoint maximising Youden’s index of 36%, the model was 86% sensitive and 88% specific. Our prevalence model was also well-calibrated, with calibration intercept = 0.051 and slope = 1 [21]. The calibration curve also showed a good correspondence between the estimated probability and the observed diagnosis of TBM (Fig. 5B). All fold-wise ROC and calibration curves showed consistent behaviour.

### Updated TBM risk if some or all confirmatory tests are negative

The changes in TBM risk relative to the pre-test values are shown in Table 3. A negative ZN-Smear alone halved the probability of TBM regardless of HIV status. A negative ZN-Smear and Xpert were associated with a 3.6-fold reduction in TBM risk for participants with HIV and a 2.4-fold reduction for those without HIV. With all tests negative, the TBM probability was reduced by 2.7 and 4.7, respectively for the two groups above.

**Table 3:** Change in probability of TBM after one or more negative confirmatory test results, relative to TBM probability when no test results are known

Scenario	Smear	MGIT	Xpert	HIV (-)	HIV ( +)
a	-	?	?	0.46 (0.25—0.62)	0.49 (0.28—0.70)
b	?	?	-	0.70 (0.58—0.79)	0.49 (0.31—0.69)
c	-	-	?	0.40 (0.19—0.56)	0.31 (0.15—0.52)
d	-	?	-	0.42 (0.21—0.58)	0.28 (0.14—0.48)
e	-	-	-	0.38 (0.18—0.54)	0.21 (0.10—0.39)

Values are in the form Median (95% Credible interval)

Assuming pre-test risk is 1, the values measure post-test risk in each scenario

In each scenario, “-” represents a negative test result and “?” represents an unknown (or unretrieved) test result

### Simplified model

**Fig. 6:**
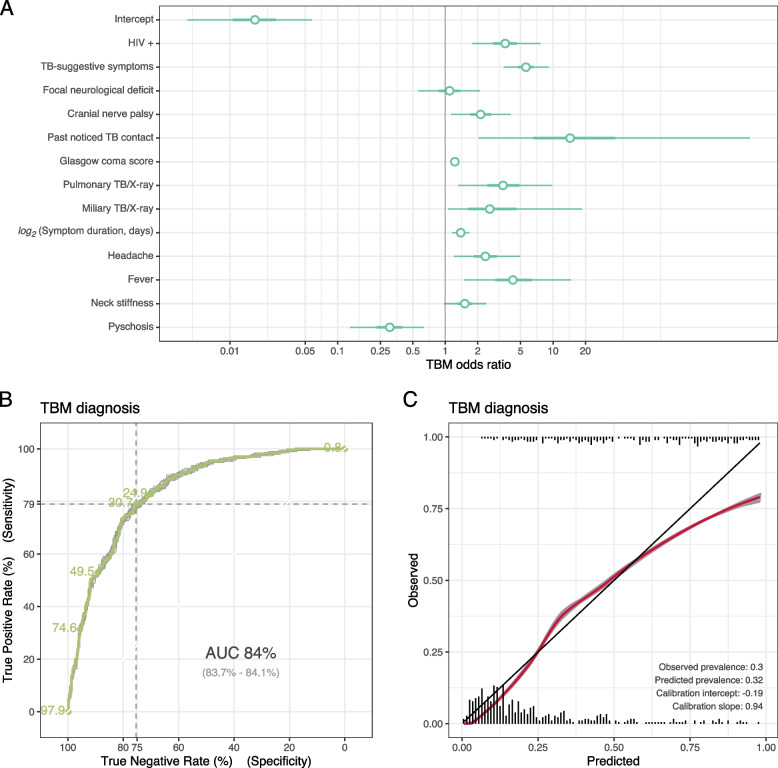
Posterior estimates and performance of the simplified prevalence model, assuming the final hospital diagnosis is the true status. **A** Posterior estimates of coefficients of clinical TBM risk factors. Points, thick and thin lines are medians, 50% and 95% credible intervals. **B** ROC plot and AUC. AUC values are presented as “average (min—max over 20 repetitions of cross-validation)”. **C** Calibration plot, showing the relationship between the predicted probability and observe outcome, smoothed by a loess curve. The grey lines are fitted curves from each 20-fold cross validation and coloured lines represents their average. The cross-validation procedure is explained in the Statistical supplementary appendix

Most included diagnostic features in the simplified prevalence model associated with TBM status (Fig. 6A). The association was not clear with focal neurological deficit. Three of the four additional symptoms suggested a TBM diagnosis, only psychosis made another diagnosis more likely.

Our simplified model had good AUC. The cutpoint maximising Youden’s index is at a probability of having TBM of 30.7%, where our model was 79.0% sensitive and 75.0% specific (Fig. 6B). We used this cutpoint to derive a screening score table by selecting rounded parameter values within the 50% credible intervals of every coefficient. All the scores and the threshold were subsequently multiplied by a factor of 2 to make the calculation easier (Table 4).

**Table 4:** TBM risk score table for screening, derived from the simplified prevalence model

Criterion	50% CrI	Value	Score
HIV	1.03—1.53	Positive	2.5
TB-suggestive symptoms	1.56—1.89	Present	3.5
Cranial nerve palsy	0.53—0.98	Present	1.5
Past noticed TB contact	1.89—3.63	Present	5.0
GCS	0.17—0.23	3–6	2.0
		7–8	3.0
		9–12	4.0
		13–15	5.0
Pulmonary TB/X-ray	0.90—1.58	Detected	2.5
Miliary TB/X-ray	0.49—1.52	Detected	2.0
Symptom duration (days)	0.27—0.39	1	0.0
		2–4	2.0
		5–7	3.0
		8–14	4.0
		15–21	5.0
		21 +	6.0
Headache	0.62—1.10	Present	1.5
Fever	1.07—1.85	Present	2.5
Neck stiffness	0.26—0.57	Present	1.0
Psychosis	-1.47—-0.93	Present	-2.5

TBM suspected: Total score >  = 14

To simplify to calculation, the estimates were doubled before choosing the scores. The decision threshold was decided by the cutpoint in the total score that maximises Youden’s Index

Continuous features were cut into bins so that each bin accounts for 1 incremental points

The model was also calibrated, with calibration slope = 0.94 and calibration intercept = -0.19. All the averaged and individual curves showed consistent behaviour (Fig. 6C), in which the estimated TBM probability corresponded well to the observed TBM diagnosis made at discharge or death.

## Discussions

We used a latent class approach to obtain a diagnostic model of TBM for adults with suspected brain infections. Upon validation, our model showed good calibration and discrimination. Using cutpoints based on Youden’s Index, both our full and simplified prevalence models surpassed the most sensitive confirmatory test ZN-Smear (86% and 79%, compared with 65% of ZN-Smear), while still obtaining good specificity (88% and 75%, respectively).

The association direction of the diagnostic features and the individual TBM risk mostly followed prior knowledge. There are two differences compared with the UCD [5]. Firstly, we enforced a positive coefficient on HIV infection. Secondly, we found that a higher GCS was associated with a higher probability of TBM. Still, our estimated TBM risk was well-calibrated with the final hospital diagnosis made at discharge or death. This raises confidence that both are accurate. However, our model provides results far earlier in patients’ hospitalisation.

A novel quantification in our model is the latent mycobacterial burden. This estimate showed good correspondence with another laboratory-based quantification (Clinical supplementary appendix). We found that higher lymphocyte count in CSF was associated with increased probability of having TBM but with reduced mycobacillary burden. As lower mycobacillary burden is believed to be associated with reduced mortality, this was in line with a previous study, in which higher lymphocyte count was linked to increased survival from TBM [22].

As a by-product, we could re-evaluate the performance of the current microbiological assays. ZN-Smear had the highest sensitivity, 64.6% (95% CrI 50.9%—81.8%), confirming prior studies from our centre and reflecting the high level of technical expertise in conducting the test [1, 11]. In our laboratory, Xpert performed poorly, especially for individuals without HIV, who on average had low mycobacillary burden. All tests performed much better for those with HIV, who tended to have higher burden. Within this group, ZN-Smear can be used as a reliable diagnostic standard, although the performance and utility are likely to be reduced outside of expert laboratories.

We are not the first to use LCA to help improve TBM diagnosis. A previous study from the Vietnam National Lung Hospital in Hanoi [6] had a different target population and estimated TBM prevalence amongst individuals with TB of any type. They made the strong assumption that all confirmatory test results were mutually independent. We could relax that assumption by including a model for mycobacterial burden. Unlike previous studies [4, 6, 8], we used non-confirmatory biomarkers as predictors, not as manifest variables. This implementation had two purposes: on the technical side, it lowered the risk of violating the aforementioned assumptions of independence if more manifest variables were included; and on the prediction side, it allowed us to develop a calibrated diagnostic model for TBM based on disease-related clinical and laboratory characteristics.

Our study has some limitations. There were missing data, especially in test results and HIV status. When imputing missing values, we had to make several assumptions. These assumptions, despite the validity checks of the imputation (Statistical supplementary appendix), could have biased the results to some extent. In addition, this is a single-centre study conducted in a specialised brain infections centre. The prevalence of individuals with severe disease may be lower in other centres. Also, although the clinicians’ s assessment and diagnosis skills were supposedly high and followed a standardised guideline, we could not rule out the risk of biases induced by personal judgement, especially in detecting clinical symptoms. The levels of clinical and laboratory expertise at our tertiary hospital may also be higher; especially the performance of CSF ZN-smear may not generalise well to other laboratories. There is less inter-laboratory variability in the performance of CSF Xpert and culture, but this does not detract from the need for external validation of our findings. In both cases, it highlights the benefit of a diagnostic tool that is less sensitive to expert judgement – which our prevalence models provide.

In conclusion, despite many past attempts to quantify microbiological test performance and develop diagnostic methods for TBM, this is amongst the first studies to utilise LCM and rigorously validate many assumptions made by the model. It was developed using a large cohort of adults with brain infection in Vietnam. Leveraging Bayesian inference, we extended the classical LCM and estimated individual mycobacillary burden. Our findings therefore have relevance for both clinical practice and research. Until a better gold standard for TBM diagnosis is developed, our model could be used as a reference for both the diagnosis of TBM and the estimation of severity, both for research and clinical care.

### Supplementary Information

**Additional file 1.** Clinical supplementary appendix.

**Additional file 2. **Statistical supplementary appendix.

## Data Availability

The datasets generated and/or analysed during the current study are available from the corresponding author upon reasonable request. All code for the actual model is available at https://github.com/trinhdhk/TBM-LCA and for the Shiny app at https://github.com/trinhdhk/Shiny.TBMLCA.

## Abbreviations

AUC: Area under the (ROC) curve
CrI: Credible interval
CSF: Cerebrospinal fluid
GCS: Glasgow coma score
HIV: Human immunodeficiency virus
HTD: Hospital for Tropical Diseases
LCA: Latent class analysis
LCM: Latent class model
MAR: Missing at random
MCAR: Missing completely at random
MGIT: Mycobacteria Growth Indicator Tube
MNAR: Missing not at random
MilTB: Miliary TB
Mtb: Mycobacterium tuberculosis
PCR: Polymerase Chain Reaction
PTB: Pulmonary TB
RGCS: Reversed Glasgow coma score
ROC: Receiver operating characteristic
SBD: Standardised bacillary burden
TB: Tuberculosis
TBM: Tuberculous meningitis
TTP: Time to positivity (for MGIT)
UCD: Uniform case definition
WCC: White cell count
Xpert: GeneXpert MTB/RIF
ZN-Smear: Ziehl-Neelsen Smear
