# Supplementary material for: A novel diagnostic model for tuberculous meningitis using Bayesian latent class analysis
Source: BMC Infect Dis. 2024 Feb 6;24:163. doi: 10.1186/s12879-024-08992-z (PMC10845506; doi:10.1186/s12879-024-08992-z)
Supplement: Supplementary file 1 — Additional file 1. Clinical supplementary appendix. [file 12879_2024_8992_MOESM1_ESM.pdf]

Clinical supplementary appendix

Contents

C.1 Latent class analysis . . . Ci
C.1.1 Motivation . . . Ci
C.1.2 Classic LCM design . . . Ci
C.1.3 Extensions to the classical LCM . . . Cii
C.2 Calculation of post-test relative TBM risk . . . Cii
C.3 Rationales for chosen methods to handle missing values . . . Ciii
C.4 Use of prior knowledge . . . Cv
C.5 Model estimates in details . . . Cvi
C.5.1 Prevalence model . . . Cviii
C.5.2 Bacillary burden model . . . Cviii
C.6 Comparison with current approaches . . . Cx
C.6.1 Comparing predicted TBM risk and the uniform case definition . . . Cx
C.6.2 Comparing the risk score and TBM risk based on the simplified prevalence model . . . Cx
C.6.3 Comparing model-based bacillary burden and current measurement . . . Cx

C.1 Latent class analysis

C.1.1 Motivation

In clinical research, logistic regression is the standard method to create a scoring system linking diagnostic features to a particular disease risk. Its reliability depends on the validity of the diagnostic gold standard. However, in the case of TBM, the uncertainty in diagnosis limits this approach. Although, positive ZN-Smear, MGIT, and Xpert are well-accepted in confirming TBM, a negative result for all three cannot rule out TBM. To address this, we utilised LCM. In this model, we examine all possible combinations of test results and identify patterns more likely to be associated with TBM. In the next section, we describe the basic structure of the model and its assumptions.

C.1.2 Classic LCM design

We describe the simplest form of our LCM and its underlying assumptions. We have 3 confirmatory test ("manifest") variables and binary disease status, all with values 0 and 1. The estimation algorithm of classical LCA starts by formulating the probability of each of the 8 patterns of manifest variables. Each probability is split up by disease status D:

$$P(T = t_1t_2t_3) = P(D = 0) \times P(T = t_1t_2t_3|D = 0) + P(D = 1) \times P(T = t_1t_2t_3|D = 1) \tag{12}$$

where d, t_1, t_2, and t_3 are either 0 or 1.

The classical LCM assumes that the results of the three tests are unrelated given disease status; they are independent in a probabilistic sense. Then the disease-specific probability of each pattern is a product of the probabilities per test (T_1, T_2, and T_3). In our specific case, (T_1, T_2, T_3) are (ZN-Smear, MGIT, and Xpert) and we have per disease status:

$$
\begin{aligned}
P(T=000|D=d) &= P(T_1=0|D=d) * P(T_2=0|D=d) * P(T_3=0|D=d)\\
P(T=001|D=d) &= P(T_1=0|D=d) * P(T_2=0|D=d) * P(T_3=1|D=d)\\
P(T=010|D=d) &= P(T_1=0|D=d) * P(T_2=1|D=d) * P(T_3=0|D=d)\\
P(T=011|D=d) &= P(T_1=0|D=d) * P(T_2=1|D=d) * P(T_3=1|D=d)\\
P(T=100|D=d) &= P(T_1=1|D=d) * P(T_2=0|D=d) * P(T_3=0|D=d)\\
P(T=101|D=d) &= P(T_1=1|D=d) * P(T_2=0|D=d) * P(T_3=1|D=d)\\
P(T=110|D=d) &= P(T_1=1|D=d) * P(T_2=1|D=d) * P(T_3=0|D=d)\\
P(T=111|D=d) &= P(T_1=1|D=d) * P(T_2=1|D=d) * P(T_3=1|D=d)
\end{aligned} \tag{13}
$$

These equations show the probability of different combinations of test results (T) given a certain disease status (D). For example, the first one shows the probability of all three test results being negative (T=000) when the disease status is d.

As all the tests are either negative or positive, $P(T_1 = 0|D = d)) = 1 - P(T_1 = 1|D = d))$, $P(T_2 = 0|D = d)) = 1 - P(T_2 = 1|D = d))$, $P(T_3 = 0|D = d)) = 1 - P(T_3 = 1|D = d))$. Let k = 1,2,3 represents the test 1,2 3, the estimates the test sensitivities $P(T_k = 1|D = 1)$, specificities $P(T_k = 0|D = 0)$ and the TBM risk $P(D = 1)$ as the values that fit the observed frequencies of the patterns.

C.1.3 Extensions to the classical LCM

C.1.3.1 Prevalence model The $P(D = 1)$ as estimated from the classical LCA is the prevalence of TBM in the study population; it does not account for individual variations in TBM risk. To better represent this variation a prevalence model has been added, in which the diagnostic features are covariables in the logistic regression as we discussed in Supplementary section C.1.1. We call this the *prevalence model* (Statistical Supplementary Section S.1.1). Somewhat simplified, the process involves a series of steps:

1. Fit the classical LCM (indicator model) to the data
2. For each individual:
   a. Use 1. to look up the corresponding probability of TBM given the pattern of test results
   b. Randomly allocate that individual to the TBM or non-TBM group according to that probability
3. Fit the logistic regression to the dataset with all individuals assigned the TBM status from 2.
4. Repeat step 2 and 3 multiple times ($\approx$ 40000 times) to get more precise results
5. Combine all the results to quantify the Bayesian posteriors of the coefficients

The additional sophistication in the algorithm is that the output from the logistic regression in step 3. is used to update individual probabilities to have TBM that are used in step 2.b.

C.1.3.2 Latent bacillary burden Classical LCM assumes that ZN-Smear, MGIT, and Xpert are independent within TBM status. This means that the mechanistic pathways ruling these tests are completely separated. This is unlikely to be true in our context. Violating the assumption can bias the result. We followed a proposed method Formula 13 and added an additional covariable to the indicator model to correct this. In case such a factor is not measured, a random effect is added. In our study, we assumed the existence of a variable that was not measured called the “mycobacillary burden” [12]. This variable might depend on other variables that are measured, which we call “modulating factors”. The details are formulated in the Statistical supplementary appendix.

C.2 Calculation of post-test relative TBM risk

The post-test relative TBM risk quantifies how much lower the risk of TBM given some or all tests have returned negative (Section 2.3). This quantification r_P is a population average and is not a replacement for individual post-test risk estimates (Formula 14). However, this can be useful, especially to justify the benefit of any repetition of the lumbar puncture. The web app [21], however, can quantify this risk on the individual level.

$$\begin{aligned} P(TBM|test) &= \frac{P(TBM) \times P(test|TBM)}{P(test)} \\ r_P = \frac{P(TBM|test)}{P(TBM)} &= \frac{P(test|TBM)}{P(test)} \end{aligned} \tag{14}$$

where $test$ is the set of available test results in each scenario (a to e). The calculation is directly derived from Bayes' rule where $P(TBM)$ is the prior probability of each individual, $P(test)$ is the conditional probability, and $P(TBM|test)$ is the posterior probability (the probability of TBM after the test).

C.3 Rationales for chosen methods to handle missing values

We handled missing data differently depending on the reason for which the data was missing. ZN-Smear, MGIT, and Xpert were not done if there was a shortage of CSF samples and TBM was unlikely. We assumed these patients were all without TBM and negative for the TBM microbiological assays as these are assumed to have high specificity [1,17]. Since HIV tests were done for all patients with suspected infection and/or involvement in high-risk activities, we could safely assume that in those who were untested, the prevalence of HIV was close to zero, as the population prevalence of HIV in Vietnam is 0.3% [32].

A summary of the suspected mechanisms of missingness and our methods to handle them is shown in Supplementary Table C1. In case a predictor is missing at random (MAR) or completely at random (MCAR), we performed a model-based imputation within the main model as part of the MCMC procedure. Further details of the imputation models are discussed in the Statistical Supplementary Section S.1.3.2.

Table C1: Rationales for chosen methods to handle missing values

**Variable**	**N missing**	**Expected Reason of Missingness**	**Mechanism**	**Handling method**
ZN-Smear	304	TBM not suspected	MNAR	Set = 0
MGIT	304	TBM not suspected	MNAR	Set = 0
Xpert	304	TBM not suspected	MNAR	Set = 0
HIV Status	211	HIV infection not suspected	MNAR	Set = 0
TB-suggestive symptoms	265	Unmeasured / Unconscious	MNAR	Imputation[1]
Focal neurological deficit	16	Unconscious	MNAR	Imputation[2]

MCAR: missing completely at random; MAR: missing at random; MNAR: missing not at random

[1] *A missing of the any compartment of TB-suggestive symtomps (Weight loss, Night sweat, and Coughing > 2 weeks) is likely a MAR given other observed compartments.*

[2] *A missing of the any compartment of Focal neurological deficit (Hemiplegia, Paraplegia, and Tetraplegia) is likely a MAR given other observed compartments.*

[3] *GCS is represented by 4 variables: E, V, M, and ReducedConsciousness. The last variable is never missing, whose value = True indicates a total GCS < maximum GCS (15 for normal cases and 10 for ventilated cases). GCS is set to 15 if ReducedConsciousness = False and not considered as missing. Otherwise, a missing of all three compartments (E, M, and V) is likely a data input error - which is MCAR - and a missing GCSV alone is likely due to ventilation and may suggest MNAR. In the latter case, we assume that we can impute GCSV based on GCSM and GCSE without bias.*

Table C1: Rationales for chosen methods to handle missing values

**Variable**	**N missing**	**Expected Reason of Missingness**	**Mechanism**	**Handling method**
Glasgow Coma Score	21	Ventilated (GCSV) / Data input error	MNAR/MCAR	Imputation[3]
Symptoms duration	26	Patient forgets / Unconscious	MAR	Imputation
Blood glucose	8	Input error / Unmeasured (premature death)	MAR/MCAR	Imputation
CSF glucose	2	Input error /Unmeasured (premature death)	MAR/MCAR	Imputation
CSF lymphocyte count	3	Implicit zero / Input error / Unmeasured (premature death)	MNAR/MAR	Manually set if implicit zero/Imputation otherwise
CSF white cell count	3	Implicit zero / Input error / Unmeasured (premature death)	MNAR/MAR	Manually set if implicit zero/Imputation otherwise
CSF protein	2	Input error / Unmeasured (premature death)	MAR/MCAR	Imputation
CSF lactate	2	Unmeasured (premature death)	MAR/MCAR	Imputation
Cryptococcal test	87	Cryptococcal meningitis not suspected	MNAR	Set = 0
Gram stain	15	Bacterial meningitis not suspected	MNAR	Set = 0
Headache	53	Reduced consciousness	MAR	Imputation

MCAR: missing completely at random; MAR: missing at random; MNAR: missing not at random

[1] *A missing of the any compartment of TB-suggestive symtomps (Weight loss, Night sweat, and Coughing > 2 weeks) is likely a MAR given other observed compartments.*

[2] *A missing of the any compartment of Focal neurological deficit (Hemiplegia, Paraplegia, and Tetraplegia) is likely a MAR given other observed compartments.*

[3] *GCS is represented by 4 variables: E, V, M, and ReducedConsciousness. The last variable is never missing, whose value = True indicates a total GCS < maximum GCS (15 for normal cases and 10 for ventilated cases). GCS is set to 15 if ReducedConsciousness = False and not considered as missing. Otherwise, a missing of all three compartments (E, M, and V) is likely a data input error - which is MCAR - and a missing GCSV alone is likely due to ventilation and may suggest MNAR. In the latter case, we assume that we can impute GCSV based on GCSM and GCSE without bias.*

Table C1: Rationales for chosen methods to handle missing values

Variable	N missing	Expected Reason of Missingness	Mechanism	Handling method
Neck stiffness	26	Reduced consciousness	MAR	Imputation
Psychosis	12	Reduced consciousness	MAR	Imputation

MCAR: missing completely at random; MAR: missing at random; MNAR: missing not at random

[1] A missing of the any compartment of TB-suggestive symtomps (Weight loss, Night sweat, and Coughing > 2 weeks) is likely a MAR given other observed compartments.

[2] A missing of the any compartment of Focal neurological deficit (Hemiplegia, Paraplegia, and Tetraplegia) is likely a MAR given other observed compartments.

[3] GCS is represented by 4 variables: E, V, M, and ReducedConsciousness. The last variable is never missing, whose value = True indicates a total GCS < maximum GCS (15 for normal cases and 10 for ventilated cases). GCS is set to 15 if ReducedConsciousness = False and not considered as missing. Otherwise, a missing of all three compartments (E, M, and V) is likely a data input error - which is MCAR - and a missing GCSV alone is likely due to ventilation and may suggest MNAR. In the latter case, we assume that we can impute GCSV based on GCSM and GCSE without bias.

C.4 Use of prior knowledge

Supplementary Table C2 displays our expected impact of diagnostic features on the individual risk of TBM. We assigned a moderate amount of prior information to the coefficients, with two exceptions. First, variables that are strongly believed to have positive/negative association with the risk were constrained to have positive/negative coefficients. The other exception was for CSF white cell count where we allowed for a quadratic relationship as suggested by prior consensus [5].

Table C2: Expected impact of demographic and clinical features on the individual TBM risk and CSF mycobacterial burden based on prior knowledge. Signs + and - in each cell indicate the expected direction of association, followed by the level of confidence - if unknown, a ? is marked, an empty cell means that we expect no association

**Predictor**	**TBM prevalence**	**Bacillary Burden**
HIV infection	+, strong	+, strong
Past TB contact	+, weak	
TB-suggestive symptoms	+, weak	
Focal neurological deficit	+, weak	
Cranial nerve palsy	+, weak	
Symptom duration	+, weak	
PTB/X-ray	+, weak	
MTB/X-ray	+, strong	
Glasgow Coma Score	-, weak	
Cryptococcus Antigen/Indian Ink	-, strong	
Positive Gram stain	-, strong	
Blood glucose	-, weak	
CSF glucose	-, weak	?, weak
CSF lymphocyte count	+, weak	-, weak
CSF white cell count	+-[1], weak	+, weak
CSF protein	+, weak	+, weak
CSF lactate	+, weak	+, weak
CSF eosinophil count	-, strong	
CSF RBC Count	?, weak	

[1] *Risk of TBM peaks with intermediate count*

For ZN-Smear, MGIT, and Xpert, we collected prior information from several previous studies [1,16,17]. A summary of these choices is shown in Supplementary Figure C1, on the linear and logistic scales. We demonstrated how well they cover corresponding results derived from the previous studies. As suggested in the literature, we used highly informative priors for False Positive Rate ($FPR = 1 - Specificity$). Given the discrepancies between the studies, we used weakly informative priors for True Positive Rate ($TPR = Sensitivity$). All are specified on the logit scale.

The details of the choice of priors are outlined in the Statistical Supplementary Section S.1.4.

C.5 Model estimates in details

This Supplementary Section outlines all the estimates of the selected model, if not mentioned in the main text.

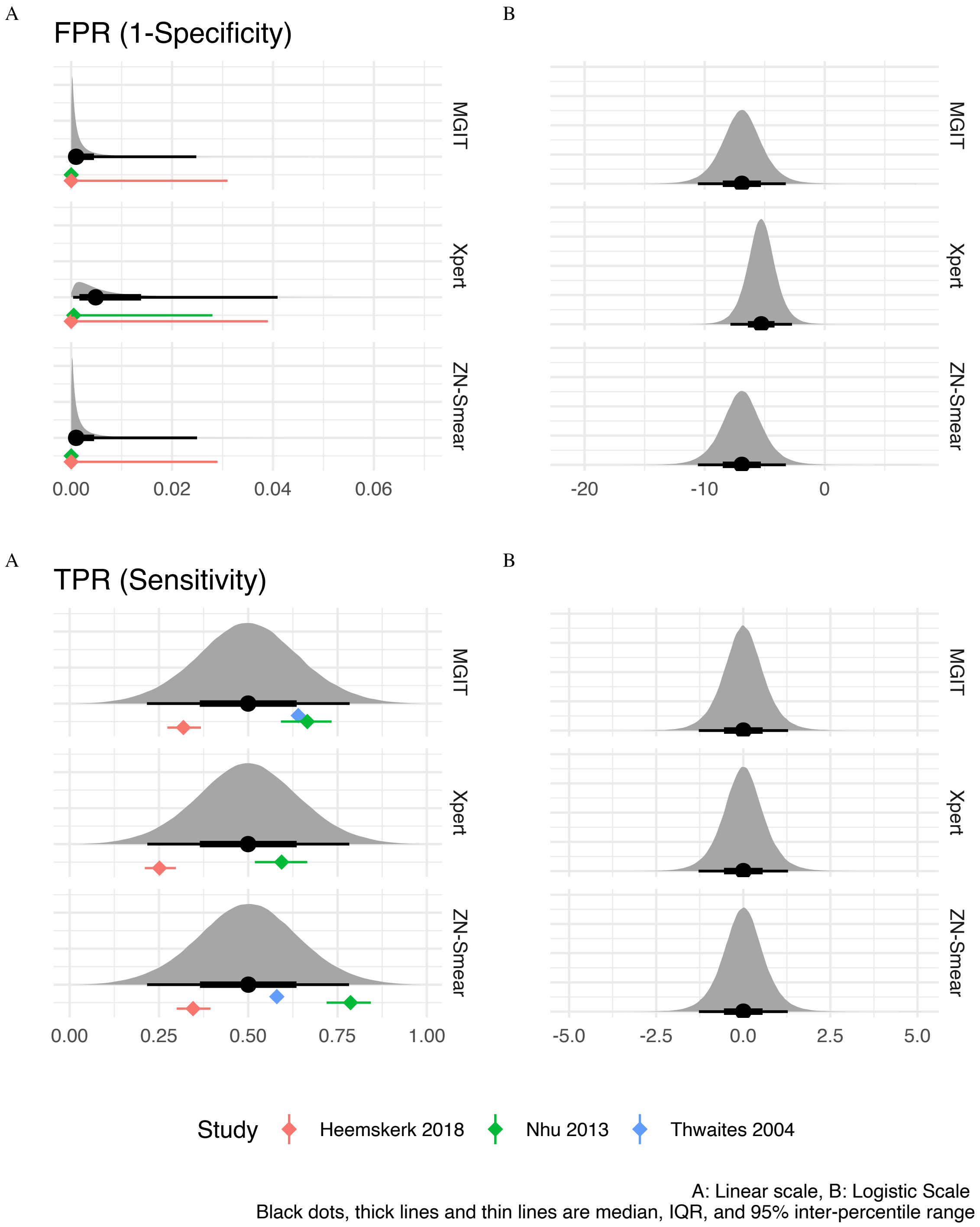

Figure C1: Density plots for prior distributions and their correspondence to prior knowledge of sensitivity and specifity of TBM confirmation tests, evaluated against then-made clinical diagnosis. Note that Thwaites 2004 was descriptive and only gave sensitivities. Specificities for ZN-Smear and Culture in Nhu 2013 were references. Hence no Confidence Intervals were given in all sensitivities reported by the former and the specificities for ZN-Smear and Culture reported by the latter.

C.5.1 Prevalence model

The medians and 95% CrIs of all estimates are reported in Supplementary Table C3, a histogram of TBM risk for each individual in the analysed sample is reported in Supplementary Figure C2, stratified by the final hospital diagnosis. In general, we saw that our prevalence model did a good job of separating the two populations where TBM (orange) cases overwhelmed the non-TBM (grey) cases in the higher-risk groups, and vice versa.

Table C3: Medians and 95% credible intervals of TBM odds ratios, with respects to diagnostic features

**Parameter**	**Median**	**95% CrI lower limit**	**95% CrI upper limit**
Intercept	0.40	0.18	1.01
HIV +	9.85	1.77	121.20
TB-suggestive symptoms	4.28	0.90	33.23
Focal neurological deficit	1.86	0.47	8.54
Cranial nerve palsy	1.15	0.29	5.04
Past noticed TB contact	5.30	0.29	7.05e+03
Glasgow coma score	1.19	0.99	1.51
Pulmonary TB/X-ray	4.05	0.66	61.46
Miliary TB/X-ray	166.22	2.84	1.39e+05
log_2 (Symptom duration, days)	1.88	1.23	3.39
log_2 (Paired blood glucose)	0.33	0.08	1.17
log_2 (CSF glucose)	0.85	0.49	1.53
log_2 (CSF protein)	1.24	0.67	2.28
log_2 (CSF lactate)	2.31	0.80	6.60
log_{10} (CSF lymphocyte)	6.35	1.19	69.10
log_{10} (CSF white cell)	2.42	0.59	13.95
log_{10} (CSF white cell)^2	0.09	0.02	0.24
CSF eosinophil > 0	6e-03	2e-05	0.13
log_{10} (CSF eosinophil)	0.35	0.02	0.95
log_{10} (CSF RBC)	0.66	0.39	0.98
Evidence of cryptococcus	3e-03	2e-05	0.05
Positive CSF Gram stain	0.02	2e-05	0.24

C.5.2 Bacillary burden model

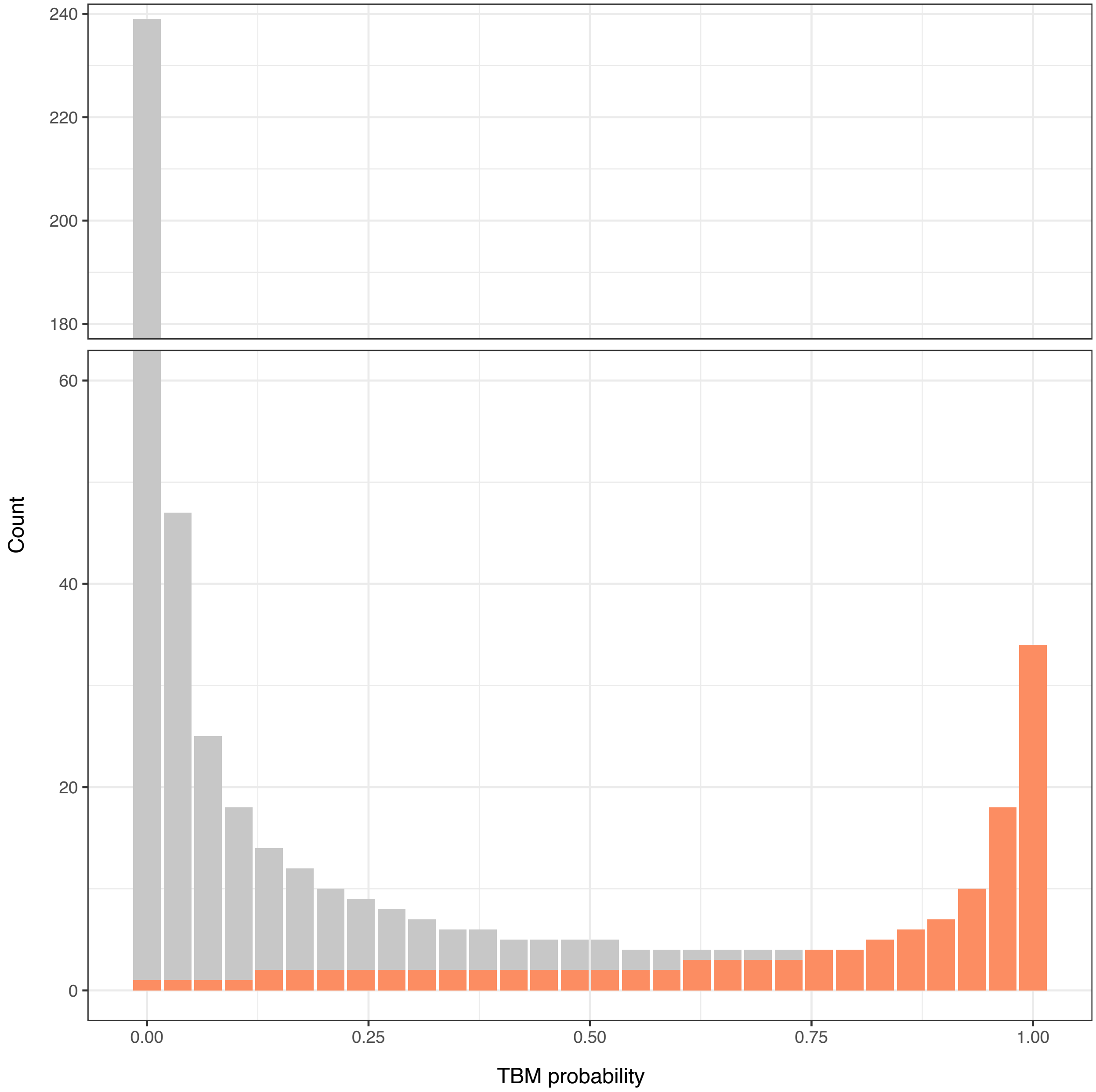

Figure C2: Histogram of individual probability to have TBM the analysed sample, as estimated by the selected model. x-axis is the TBM probability, and the count on the y-axis is the estimated number of individuals in each bin. Microbiologically confirmed cases are coloured in orange.

Table C4: Estimates and 95% credible intervals of standardised bacillary burden, with respects to modulating factors

Parameter	Median	95% CrI lower limit	95% CrI upper limit
HIV +	1.51	0.83	2.28
GCS	-0.16	-0.29	-0.04
log_2 (CSF glucose)	-0.35	-0.76	0.06
log_2 (CSF protein)	1.07	0.53	1.73
log_2 (CSF lactate)	0.06	-0.55	0.70
log_{10} (CSF lymphocyte)	-0.53	-1.25	0.11
log_{10} (CSF white cell)	-0.20	-0.49	0.10

C.6 Comparison with current approaches

C.6.1 Comparing predicted TBM risk and the uniform case definition

In this section, we compared the prediction of our selected model and the standard TBM uniform case definition [5] (Supplementary Figure C5). We excluded the results from the confirmatory tests in the score. Visually, there was a positive correlation between the two, with patients with lower scores having lower mean TBM probability. However, without the definitive microbacterial tests, the uniform case definition missed out on some confirmed cases as they were classified into the unknown group (or undefined group, with a total score < 6). An interesting phenomenon was that the correlation is not monotonous. In our dataset, patients with score < 6 had the same risk as those who have a score 5-6 points higher (for instance, those scoring 4 had apparently similar risk distribution as those scoring 9).

C.6.2 Comparing the risk score and TBM risk based on the simplified prevalence model

The screening risk score (Table 4) is a direct derivation from the simplified prevalence model. This is a manual task that involves choosing a point estimate from within the 50% CrI for every coefficient, on the original scale of the corresponding covariable. The threshold (6) was calculated as the log-odd of the optimal cutpoint (28.2%) and after correction for the standardisation of diagnostic feature values. In this section, we compared the risk score calculated from this table with the actual probability of TBM estimated by the simplified prevalence model (Supplementary Figure C3) and how the risk score would perform on hospital diagnosis (Supplementary Figure C4). In Table 4, the total point of 14 was chosen as the optimal threshold by maximising Youden's J Index, however, a threshold of 13 points can be considered if sensitivity is in favour.

C.6.3 Comparing model-based bacillary burden and current measurement

In this section, we compared our estimated *standardised mycobacillary burden* (SMB) with two tools to quantify bacterial load. These tools are the semi-quantification level by GeneXpert^{TM} (based on CT value) and *time to positivity* (TTP) when performing culturing. The general downside of these quantification methods is that they require the corresponding tests to be positive. As a standard rule, clinicians assume negative patients to have low bacterial load.

We can see a positive correlation between our SMB and the Xpert level (Supplementary Figure C6). The correlation was not obvious between SMB and TTP in the overall population. This might be due to small sample size. However, similar to a previous study [33], in the sub-population without HIV, we can see a negative correlation between TTP and SMB; that was however not obvious in the other group (Supplementary Figure C7). The association was again supported by the linear regression between TTP and SMB, correction

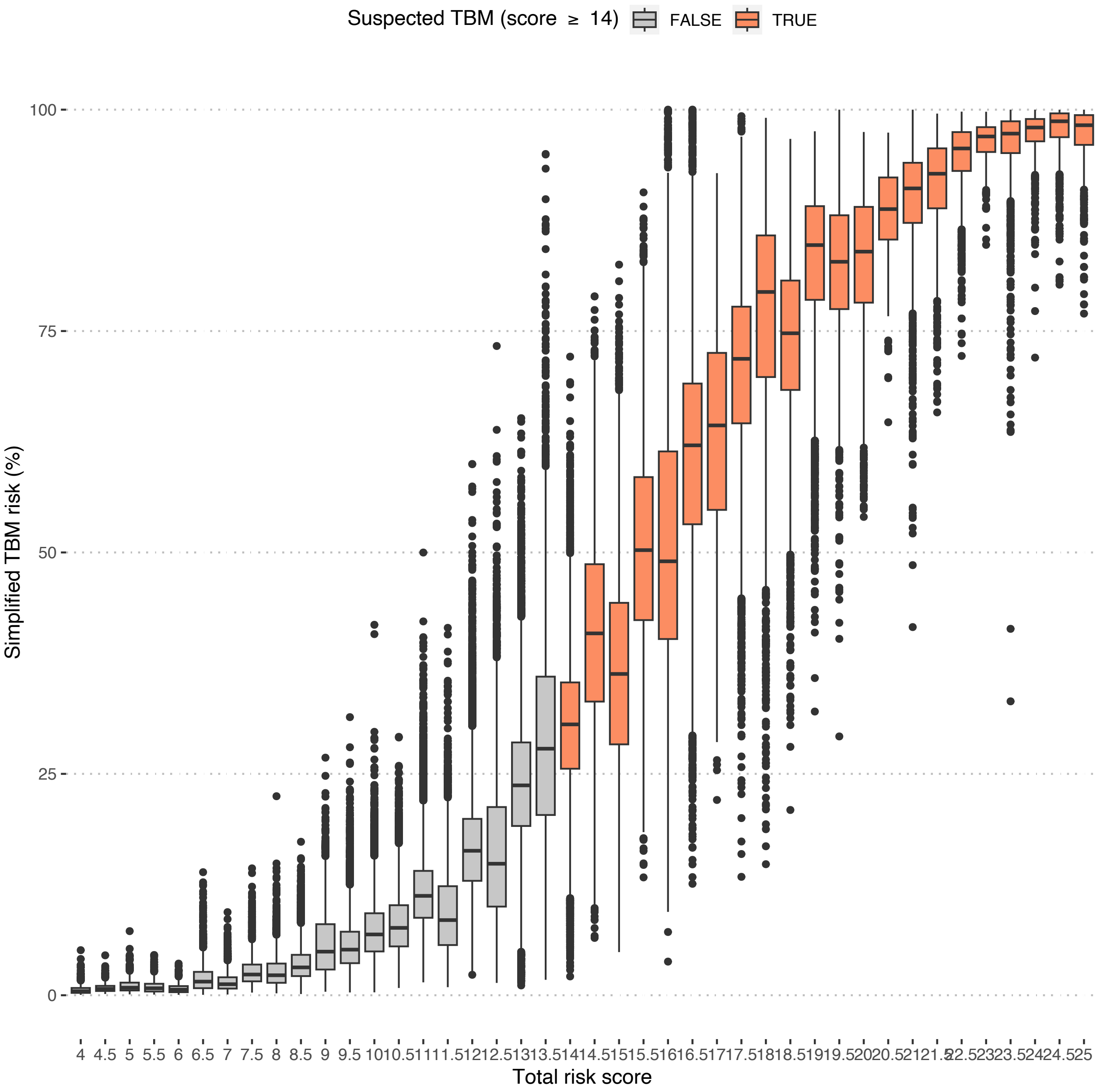

Figure C3: Comparison of the TBM screening risk score and the TBM probability based on the simplified prevalence model

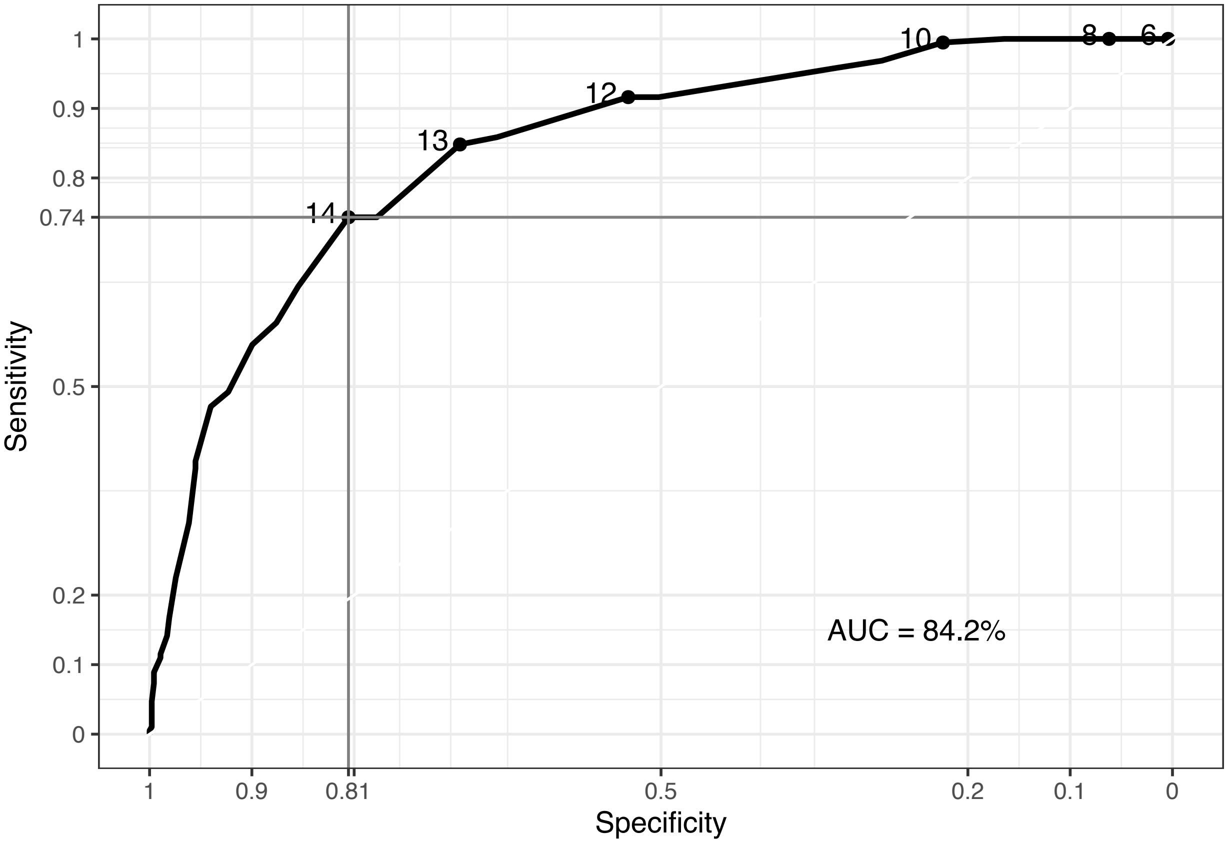

Figure C4: ROC curve of the screening risk score against the final hospital diagnosis

for HIV status and the interaction between TTP and HIV. The negative association was nullified in sub-population with HIV (Supplementary Table C5). This contradicted the past study [33] and possibly due to the low number of TBM-HIV co-infections that had a positive MGIT culturing.

Table C5: Comparison of our predicted SMB and TTP

**Characteristic**	**Beta**	**95% CI**[1]	**p-value**
HIV	-2.5	-6.7, 1.7	0.2
log(TTP)	-0.43	-0.81, -0.05	0.026
HIV * log(TTP)	0.43	-0.07, 0.93	0.093

[1] CI = Confidence Interval

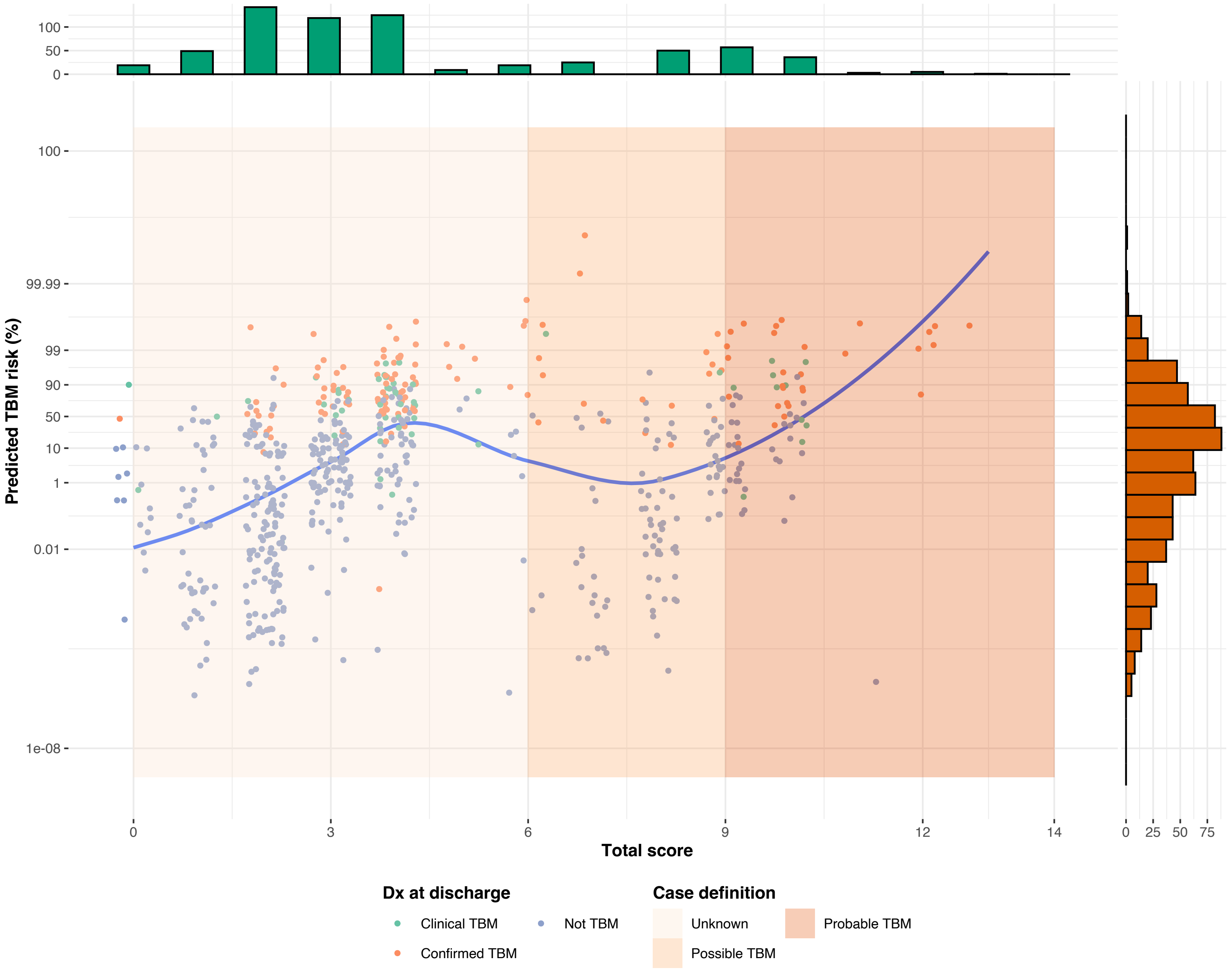

Figure C5: Comparison of our predicted TBM probability and the calculated scores and classes by the uniform definition

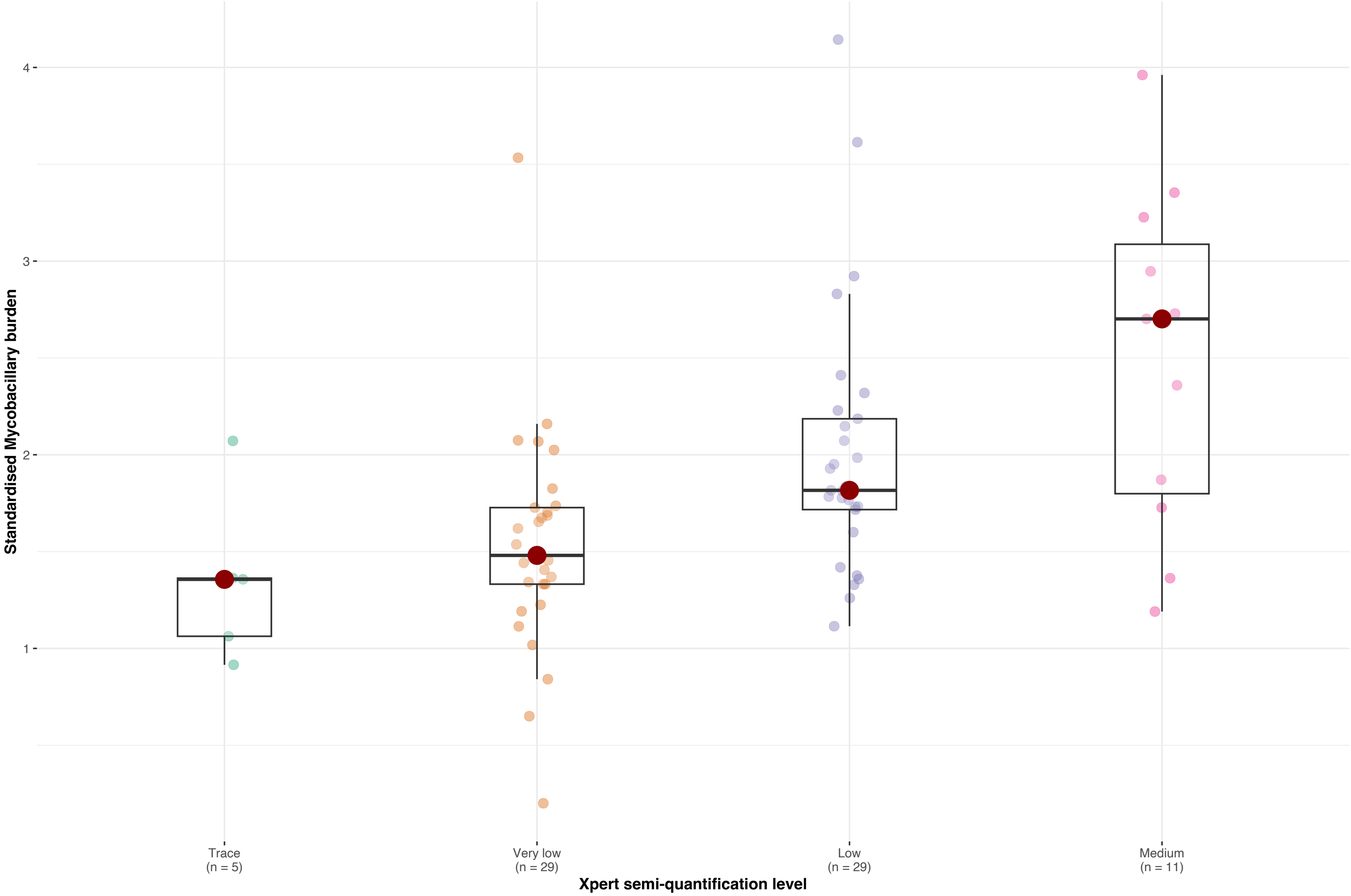

Figure C6: Comparison of our predicted SMB and Xpert semi-quantification level

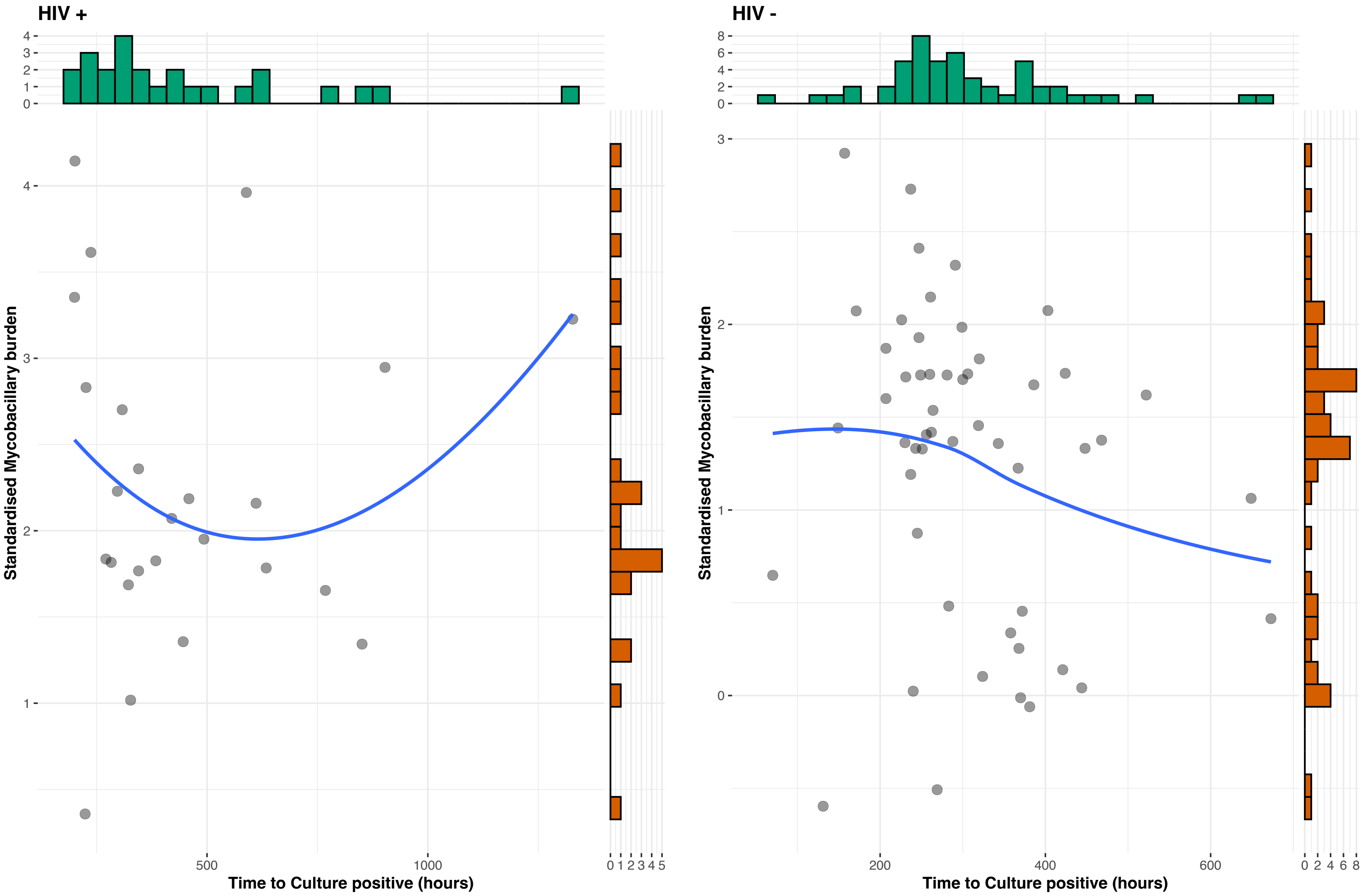

Figure C7: Comparison of our predicted SMB and TTP
