# Supplementary material for: A novel diagnostic model for tuberculous meningitis using Bayesian latent class analysis
Source: BMC Infect Dis. 2024 Feb 6;24:163. doi: 10.1186/s12879-024-08992-z (PMC10845506; doi:10.1186/s12879-024-08992-z)
Supplement: Supplementary file 2 — Additional file 2. Statistical supplementary appendix. [file 12879_2024_8992_MOESM2_ESM.pdf]

Statistical supplementary appendix

Contents

S.1 Model Specification Details . . . . Si
S.1.1 Mathematical parametrisation . . . . Si
S.1.2 Simplified model . . . . Siii
S.1.3 Preprocessing and imputation . . . . Siii
S.1.4 Choice of priors . . . . Sv
S.2 Model diagnosis and comparison . . . . Sviii
S.2.1 Estimated log-pointwise density of each considered model . . . . Sviii
S.2.2 Sampling diagnosis for the selected model . . . . Sviii
S.2.3 Perfomance validation of the selected model on observed manifest variables (i.e. mycobacterial tests) . . . . Sx
S.2.4 Scaled Brier scores of the prevalence models against the final hospital diagnosis . . . . Sx
S.3 Assumption checks and sensitivity analysis . . . . Sxi
S.3.1 Methods . . . . Sxi
S.3.2 Results . . . . Sxiii
S.4 Analysis session info . . . . Sxv

S.1 Model Specification Details

S.1.1 Mathematical parametrisation

Our model consists of two components:

1. *Prevalence model*: A logistic regression model that quantifies the probability of having TBM (θ) based on a set of diagnostic features (X):
$$\text{logit}(\theta) = X^T\alpha$$
where X and α are vectors of covariate values and coefficients.

2. *Indicator model*: three logistic regression models, one for each confirmatory test, that quantify the probability of having a positive test result depending on TBM status and additional variables as described in Supplementary Table S1. Similar to previous studies [7,12,24], we allowed the test result to depend on the unobserved bacillary burden and systematic noise of the procedure. Both were in turn regressed on relevant biomarkers and an unobserved random effect. Presumably, TBM patients with lower bacillary burden are less likely to test positive. Our models 1,2 and 5 are similar in spirit to models M1, M3 and M4 in Schumacher *et al.* [7]. In their models, they did not make the direct link between the covariates and bacillary burden, which is what we do in our models 3 and 4. These two models are in-between M3 and M4.

We give a detailed description of the structure of our indicator models in Supplementary Table S1. For $1 \leq i \leq 659$, let $C_i \in \{0, 1\}$, and $\theta_i = P(C_i = 1)$ be the *true TBM status* and the *probability of having TBM*; $y_i^{ZN\text{-}Smear}$, y_i^{MGIT}, and y_i^{Xpert} are the *observed test outcomes*. The joint distribution of the observed test results is:

$$\begin{aligned} P_i &= P(y_i^{ZN\text{-}Smear}, y_i^{Mgit}, y_i^{Xpert}) \\ &= \theta_i \times P(y_i^{ZN\text{-}Smear}, y_i^{Mgit}, y_i^{Xpert} | C_i = 1) \\ &\quad + (1 - \theta_i) \times P(y_i^{ZN\text{-}Smear}, y_i^{Mgit}, y_i^{Xpert} | C_i = 0) \end{aligned} \tag{1}$$

Under the assumption that test results are mutually independent given TBM status, we obtained the likelihood function for Model 1. The contribution to the likelihood for individual i is:

$$P_i = \theta_i \times P(y_i^{ZN\text{-}Smear}|C_i = 1) \times P(y_i^{Mgit}|C_i = 1) \times P(y_i^{Xpert}|C_i = 1) + (1 - \theta_i) \times P(y_i^{ZN\text{-}Smear}|C_i = 0) \times P(y_i^{Mgit}|C_i = 0) \times P(y_i^{Xpert}|C_i = 0) \tag{2}$$

This independence assumption is likely to be too strong because all tests are based on the detection of *Mtb* in the CSF. Therefore, we introduced a latent variable, denoted as bacillary burden. For models 2 and 3, we assumed that the test results were independent conditionally on TBM status and bacillary burden (B) [7]. Individuals without TBM have zero bacillary burden by nature. The contribution to the likelihood, conditionally on bacillary burden B_i, becomes:

$$\begin{aligned} P_i|B_i &= P(y_i^{ZN\text{-}Smear}, y_i^{Mgit}, y_i^{Xpert}|B_i) \\ &= \theta_i \times P(y_i^{ZN\text{-}Smear}, y_i^{Mgit}, y_i^{Xpert}|C_i = 1, B_i) \\ &\quad + (1 - \theta_i) \times P(y_i^{ZN\text{-}Smear}, y_i^{Mgit}, y_i^{Xpert}|C_i = 0) \\ &= \theta_i \times P(y_i^{ZN\text{-}Smear}|C_i = 1, B_i) * P(y_i^{Mgit}|C_i = 1, B_i) \times P(y_i^{Xpert}|C_i = 1, B_i) \\ &\quad + (1 - \theta_i) \times P(y_i^{ZN\text{-}Smear}|C_i = 0) * P(y_i^{Mgit}|C_i = 0) \times P(y_i^{Xpert}|C_i = 0) \end{aligned} \tag{3}$$

We defined Bacillary burden as a unitless, standardised variable (i.e., following a Standard Normal distribution), which in turn depends on a set of features (denoted as modulating factors V_i) via a linear regression. We used a mixed-effects logistic regression to capture the association between B and test results ($y^{(t)}$). In model 3 we assumed:

$$\begin{aligned} \text{logit}[P(y_i^{(t)}|C_i = 1, B_i)] &= z_0^{(t)} + B_i \times \beta^{(t)} \\ &= z_0^{(t)} + (V_i^T\gamma + r_i)\beta^{(t)} \end{aligned} \tag{4}$$

where $(t) \in$ {ZN-Smear, Mgit, Xpert}, $z_0^{(t)}$ is the intercept, and $\beta_B^{(t)}$ is the coefficient of bacillary burden on the result of test (t), γ is the vector of coefficients for V_i, and the individual random effect r_i quantifies the fluctuation of bacillary burden not explained by V_i. V_i and r_i are assumed to be independent of each other. Model 2 is a special case of model 3 in which the $\beta^{(t)}$ are equal over (t), which is the same as Model M3 in Schumacher *et al.* [7]. Model 3 assumes that for each pair of modulating factors, although their impact on test results (as expressed by $\beta^{(t)}$) could differ by test, their relative contribution (as measured by the pairwise ratios of their corresponding parameters) is constrained to be identical:

$$\frac{\gamma^{[k]}\beta^{(t)}}{\gamma^{[l]}\beta^{(t)}} = \frac{\gamma^{[k]}}{\gamma^{[l]}} \tag{5}$$

for each two elements $\gamma^{[k]}$ and $\gamma^{[l]}$ of vector γ.

In model 4, we additionally captured the sample-wise fluctuation not explained by the bacillary burden (Formula 6). This fluctuation was modelled by one extra standardised random effect (u_i), with coefficient $\upsilon^{(t)}$ serving as the standard deviation of u_i. Contrary to r_i and $\beta^{(t)}$, it was not related to the modulating factors V_i and was assumed unique for each individual. We used penalisation on $\upsilon^{(t)}$ and $\beta^{(t)}$, as specified via the prior.

$$\begin{aligned} \text{logit}[P(y_i^{(t)}|C_i = 1, B_i)] &= z_0^{(t)} + B_i \times \beta^{(t)} + u_i\upsilon^{(t)} \\ &= z_0^{(t)} + (V_i^T\gamma + r_i)\beta^{(t)} + u_i\upsilon^{(t)} \end{aligned} \tag{6}$$

In model 5 (Formula 7), we explored all potential impacts of V_i to test results by linking them directly to $P(y_i^{(t)})|C_i = 1, V_i)$. This is the most flexible design and most similar to model M4 [7]. It doesn't constrain the relative contribution of each pair of modulating factors to be the same for all tests as in Formula 5.

$$\text{logit}[P(y_i^{(t)}|C_i = 1, V_i)] = z_0^{(t)} + V_i^T \gamma^{(t)} + \upsilon^{(t)} r_i \tag{7}$$

Table S1: Different model designs

Model	**Base description (Only additional effects compared to the preceding number are mentioned)**
1	No bacillary burden; everyone in the same TBM class has equal risk to test positive
2	Test results depend on individual bacillary burden; impact of bacillary burden on test result is the same across all tests
3	Impact of bacillary burden differs between tests
4	Added technical fluctuation as a second random effect; fixed effects only contribute to bacillary burden
5	Modulating factors also contribute to technical fluctuation (i.e. direct associations between V and P(Y), not mediated by mycobacillary burden)

Posterior distributions were estimated using 4 chains, each with 2000 warm-up and 8000 effective iterations with a thinning of 2 (i.e., keeping every second iteration).

S.1.2 Simplified model

The simplified prevalence model only includes diagnostic features for which a lumbar puncture is not needed. We didn't fit another latent class model, because it is likely to give worse TBM allocation than the LCM based on the full set of variables. Instead, we generated the response variable $\hat{C}_i \in \{0, 1\}$ from the posterior distribution of the individual probability θ_i to have TBM as fitted from the best performing model in Supplementary Table S1 (see Supplementary Table S3). The generated values were regressed on the restricted set of diagnostic features K. Hence:

$$\hat{C}_i \sim Bernoulli(\hat{\theta}_i)$$
$$\text{logit}[P(\hat{C}_i = 1)] \sim K_i^T \kappa$$

where κ is the vector of coefficients for the covariates K

To do this in `Stan`, we first generated 400 sets of individual TBM statuses $\hat{C}_1, \dots \hat{C}_{659}$ from the posterior distribution of the selected LCM. Then we related each set to the diagnostic features (with missing values completed in the best-performing LCM model in Supplementary Table S1). Per set, we ran one MCMC chain with 2000 warm-up and 4000 effective iterations. To reduce the memory footprint, we thinned the chains by 40. Finally, we combined all these chains for posterior summaries.

S.1.3 Preprocessing and imputation

S.1.3.1 Data preprocessing The choice of diagnostic features X and modulating factors V was inspired by the uniform case definition [5]. Prior knowledge of different predictors is shown in Supplementary Table C2. Symptom duration and CSF biomarkers were transformed to a logarithmic scale as they were right-skewed. We followed Gelman's scale-matching method [25], in which continuous predictors were centred by their empirical means and subsequently scaled by 2 times their standard deviations (sd). Binary features were kept as is. Since CSF eosinophil count was heavily zero-inflated, we created an extra indicator for count > 0 and rescaled the positive values by their sd. Glasgow Coma Score (GCS) and its components (Voice -

GCSV, Eyes - GCSE, and Muscle - GCSM) were translated to *Reversed GCS* (RGCS = RGCSV + RGCSE + RGCSM) so that a $GCS = 15$ would be equivalent to $RGCS = 0$, while $GCS = 3$ would be translated to $RGCS = 12$ (Supplementary Table S2). This transformation facilitated the choice of prior in the imputation process as all of them shared the minimum of 0 for the healthy condition (instead of starting at arbitrary numbers) and also improved numerical stability during sampling. We scaled down the auxiliary variables RGCSE, RGCSM, and RGCSV to the range $[0, 1]$. Hence $RGCS = 3RGCSE + 4RGCSV + 5RGCSM$. Binary variables were dummy-coded into 0 for "Negative" / "No" and 1 for "Positive" / "Yes".

Table S2: Conversion table from classical Glasgow coma scales to Reversed Glasgow coma scales

**Feature**	**Response**	**GCS**	**RGCS**
Eye response	Open spontaneously	4	0
	Open to voice command	3	1
	Open to pain	2	2
	No eye open	1	3
Verbal response	Orientated	5	0
	Confused	4	1
	Inappropriate words	3	2
	Incomprehensible sounds	2	3
	No verbal response	1	4
Motor response	Obey command	6	0
	Localising pain	5	1
	Withdrawal from pain	4	2
	Flexing	3	3
	Extending	2	4
	No motor response	1	5

S.1.3.2 Imputation strategy Our imputation models are summarised in Supplementary Figure S1, together with the variables that were used as regressors. Imputation was not done separately but as part of the MCMC sampling process. Potentially correlated variables were grouped together and sampled from a multivariate distribution. Numerical features were imputed using Multivariate linear regression:

$$X_{cont} \sim \mathcal{N}(L^T\psi, \Sigma) \tag{8}$$

where X_{cont} is the vector of continuous variables with missing values, and L^T is the vector of regressors used in the respective imputation model, with coefficients ψ. $\mathcal{N}$ is the Normal distribution in univariate case and Multivariate Normal distribution otherwise with Σ as the variance-covariance matrix. Composite variables (*TB-suggestive symptoms*, *focal neurological deficit*, and *RGCS*) were imputed via their corresponding compartments.

For *headache*, *neck stiffness*, and *psychosis* (Supplementary Table C1), which only appear in the simplified prevalence model, we used predictive mean matching [26]. The imputation is done once for each of the 400 generated datasets with TBM statuses. Their donors are other completed variables imputed within the full model.

S.1.4 Choice of priors

S.1.4.1 Main model For the prevalence model, we chose a Student's t distribution with 6 degrees of freedom (t_6) for the intercept, in which we fixed the mean to 0 and scale to 3 because its absolute value is unlikely to be higher than 6 (which is equivalent to a probability range of (0.002 - 0.998)) [27]. For the regression coefficients, we used t_6 distributions with mean set to 0 and for scales we chose a prior $Half\text{-}Normal(0, 1.2)$. This acted as a shrinkage term, reflecting our belief that the absolute values of the

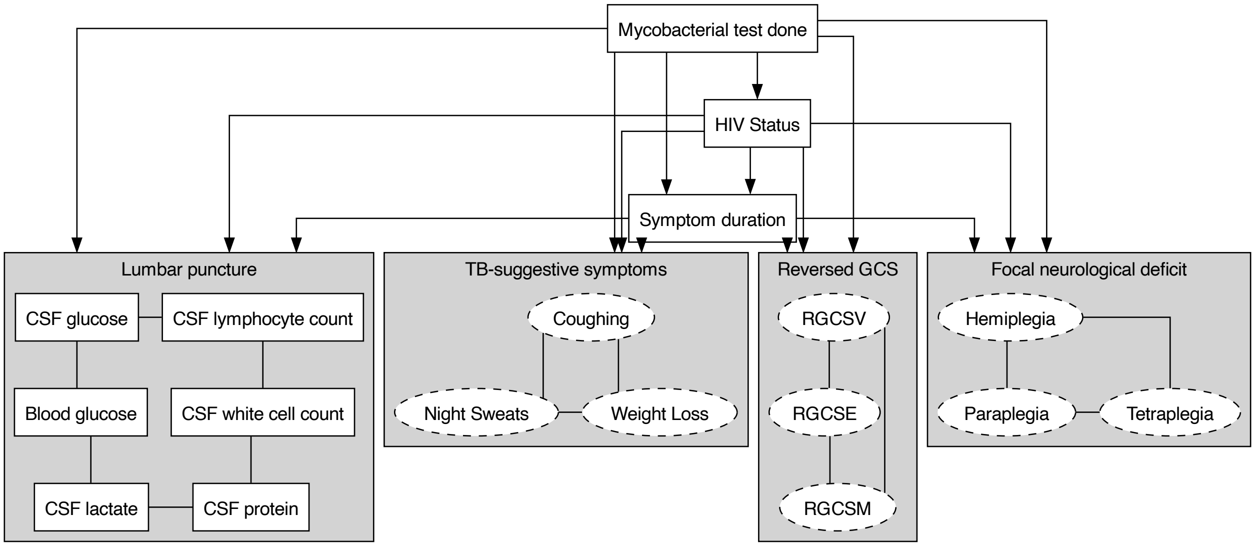

Figure S1: Imputation strategy for variables with missing values. Variables contributing to the LCM are in white boxes with solid borders. Variables in ovals with dashed borders were used in the imputation model. They make up the composite variables that were used in the LCM (mentioned in the top of the grey boxes). Variables in a grey box were imputed together via a multivariate regression. Arrows demonstrate covariate → response relations. "Mycobacterial test done" is defined as "ZN-Smear, MGIT and Xpert were performed on the patient's CSF sample"

coefficients are smaller than 4.8 (which is equivalent to a range of 0.008 - 121 for odds ratio) [28,29], but we left some room for our belief to be wrong. Hence we chose:

$$
\begin{aligned}
\alpha_0 &\sim t_6(0, 3) \\
\alpha_{binary} &\sim t_6(0, \sigma) \\
\alpha_{continuous} &\sim t_6(0, \frac{\sigma}{2sd}) \\
\sigma &\sim Normal(0, 1.2)\mathsf{T}[0, \infty)
\end{aligned}
$$

where $\mathscr{D}\ \mathsf{T}[0, \infty)$ is the distribution $\mathscr{D}$ truncated to the non-negative half line; σ is the adaptive penalty term; α_0, α_{binary}, and $\alpha_{continuous}$ are the intercept, coefficients for the binary covariates, and coefficients for the continuous covariates included in the prevalence model; sd is the empirical standard deviation of the continuous variables after the imputation and differs by each variable. For high-impact diagnostic features (marked as "strong" in Supplementary Table C2), priors on the parameters were truncated at 0, so that only values in the expected direction were accepted in the MCMC chains.

For the indicator model, our choice of priors was based on information collected from previous studies [1,16,17]. A visual justification is given in Supplementary Figure C1. As suggested by the TBM literature, we used highly informative priors for false positive rate (FPR = 1−**Spec**ificity) and weakly informative priors for true positive rate (TPR = **Sens**itivity), given the discrepancies in TPR between the studies (Formula 9).

$$
\begin{aligned}
1 - \text{Spec}_{Xpert} &\sim Logistic(\text{logit}(0.005), 0.7) \\
1 - \text{Spec}_{MGIT} &\sim Logistic(\text{logit}(0.001), 1) \\
1 - \text{Spec}_{ZN\text{-}Smear} &\sim Logistic(\text{logit}(0.001), 1) \\
\text{Sens}_{Xpert},\ \text{Sens}_{MGIT},\ \text{Sens}_{ZN\text{-}Smear} &\sim Logistic(0, .5)
\end{aligned}
\tag{9}
$$

For coefficients γ that form part of the modulating factor sub-model, we applied the same rules as in the

prevalence model. Both random effects r and u were assumed to follow a Standard Normal distribution. To avoid sign switching, we constrained $\beta^{(t)}$ and $v^{(t)}$ to be non-negative. As coefficients γ and $\beta^{(t)}$ were multiplied together, to provide a fair penalisation, we divided the penalty term σ' for γ by the average of $\beta^{(t)}$ over the three tests t = *{ZN-Smear, Mgit, Xpert}*.

Hence we chose

$$
\begin{aligned}
r &\sim Normal(0,1)\\
u &\sim Normal(0,1)\\
\sigma' &\sim Normal(0,1)\\
\beta^{(t)} &\sim Normal(0,\sigma')\mathsf{T}[0,\infty)\\
v^{(t)} &\sim Normal(0,\sigma')\mathsf{T}[0,\infty)\\
\gamma_{HIV} &\sim t_6(0,\sigma'/\overline{\beta^{(t)}})\\
\gamma_{continuous} &\sim t_6(0,\frac{\sigma'}{2sd\times\overline{\beta^{(t)}}})
\end{aligned}
$$

where γ_{HIV} is the coefficient for HIV status; $\gamma_{continuous}$ are the coefficients of CSF biomarkers; σ' is the penalty term for the bacillary burden model; $\overline{\beta^{(t)}}$ is the average of $\beta^{(t)}$ over (t); sd is the empirical standard deviation of each continuous variable after the imputation and differs per variable.

S.1.4.2 Imputation model For the imputation models, $Normal(0,2.5)$ was chosen as prior distributions for intercepts and coefficients [28], except for RGCS (E, V, and M) as their values are constrained to be between 0 and 1 (Section S.1.3.1). Their modes hence were sampled from an $DoubleExponential(0,0.05)$, and $Normal(0,0.25)$ was chosen as the prior distribution for their sd.

$$
\begin{aligned}
\psi_0 &\sim Normal(0,2.5)\\
\psi &\sim Normal(0,2.5)\\
\mathcal{G}(X_{imp}) &\sim \mathcal{D}(\psi_0+L^T\psi,\Sigma)
\end{aligned}
$$

$$
\begin{aligned}
\psi_{RGCS_0} &\sim DoubleExponential(0,0.05)\\
\psi_{RGCS} &\sim Normal(0,0.25)\\
RGCS &\sim Normal(\psi_{RGCS_0}+L^T\psi_{RGCS},\Sigma_{RGCS})\mathsf{T}[0,1]
\end{aligned}
$$

where ψ_0, ψ, and L are the intercept, coefficients, and regressors involved in each imputation model of X_{imp}. $\mathcal{G}$ is the link function (logit if X_{imp} is binary and identity otherwise). In the univariate cases, $\mathcal{D}$ is the univariate Logistic distribution if X_{imp} is binary and Normal distribution otherwise; Σ is the standard deviation. In the multivariate cases, $\mathcal{D}$ is the corresponding multivariate version and Σ is the Cholesky decomposition of the variance-covariance matrix. This matrix is the multiplication of the diagonal matrix Diag(σ) of the standard deviations and the corresponding Choleskly decomposed correlation matrix Ω. In that context, Ω was sampled from a Cholesky Lewandowski-Kurowicka-Joe (LKJ) Correlation prior with $\eta = 4$ [19].

$$
\begin{aligned}
\Sigma &= \mathrm{Diag}(\sigma)\Omega\\
\Omega &\sim LKJ(\eta=4)\\
\sigma &\sim Normal(0,1)\mathsf{T}[0,\infty)\\
\sigma_{RGCS} &\sim Normal(0,0.1)\mathsf{T}[0,\infty)
\end{aligned}
$$

S.2 Model diagnosis and comparison

For each parameter, we evaluated the Brooks-Gelman-Rubin $\hat{R}$ statistic, effective sample size per individual and Monte Carlo standard errors.

Model performance was estimated and compared using expected log pointwise density (elpd) [20]. This measure quantifies the likelihood of a new observation (y_{new}) given the data y, model $\mathcal{M}$, and posterior distributions of the parameters θ. In our case, (y_{new}) are the held-out observations using cross-validation (see below). A larger elpd means that the predicted posterior distribution concentrates around the observed response values.

All elpd, ROC curves, AUCs, and calibration metrics are calculated using 5 repetitions of 20-fold cross-validation. For each repetition, we pooled all held-out folds together to reconstruct a complete dataset. The models were fitted on the complements of the held-out folds. The averaged performance measures were acquired by pooling all held-out folds over the 5 repetitions.

The cross-validation procedure for the simplified model was done in the same manner, but adjusted for the fact that the outcome $\hat{C}_i$ was generated from the posterior θ_i of the disease prevalence; we simulated a different response vector $\hat{C}_i^k$ (of size 400) for every fold k in each repetition i in which $1 \leq k, i \leq 20$. The final AUC, ROC, and calibration were averaged over these $20 \times 20 = 400$ experiments.

As AUCs and calibrations of the selected model are reported in the main text, here we only report the results of the elpd measure for model comparison.

S.2.1 Estimated log-pointwise density of each considered model

Table S3: Expected elpd of considered models and their standard errors. Model 3 and 4 have comparable elpd. Model 3 is selected because it is simpler.

Model	elpd
1	-377.01
2	-352.60
3	-346.54
4	-347.04
5	-353.34

S.2.2 Sampling diagnosis for the selected model

Supplementary Figure S2 [19] shows the histogram of the Brooks-Gelman-Rubin $\hat{R}$ statistic, effective sample size, and Monte Carlo standard errors over posterior standard deviation (SD) across all parameters. A good mixing between chains should have $\hat{R}$ near 1. A Monte Carlo standard error over posterior SD near 0 means not much uncertainty in the posterior estimate comes from the MCMC sampling process. The effective sample size measures the equivalent number of independent samples from the posterior distribution our MCMC sampling process represents. As a rule of thumb, an effective sample size of at least 400 is sufficient to approximate the true distribution [19]. Overall, no signs of poor chain mixing and unreliable sampling of the posteriors were suggested by the diagnostics plots, with most $\hat{R}$ highly concentrated around $1 \pm .005$ and effective sample size $\geq 50\%$ of the number of samples (16000 for the full model, and 40000 for the simplified model).

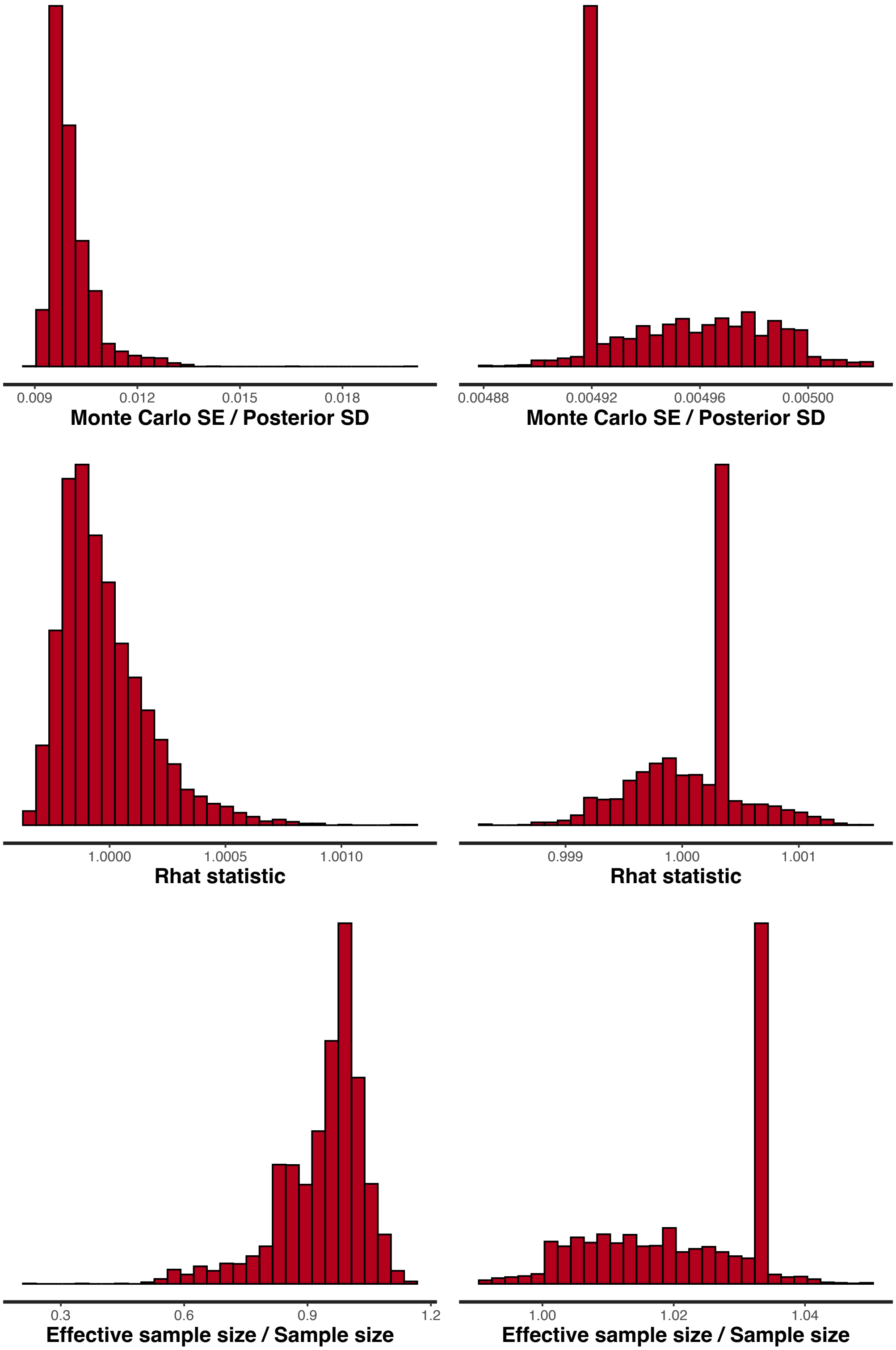

Figure S2: Sampling diagnostic statistics for the selected full model (LHS) and the simplified prevalence model (RHS)

S.2.3 Perfomance validation of the selected model on observed manifest variables (i.e. mycobacterial tests)

As mentioned in the main text, the prevalence model was pseudo-validated on the final hospital diagnosis. Here we performed a proper validation of the selected model on the observed manifest variables (Supplementary Figure S3) demonstrating the performance of the selected model against the observed mycobacterial tests.

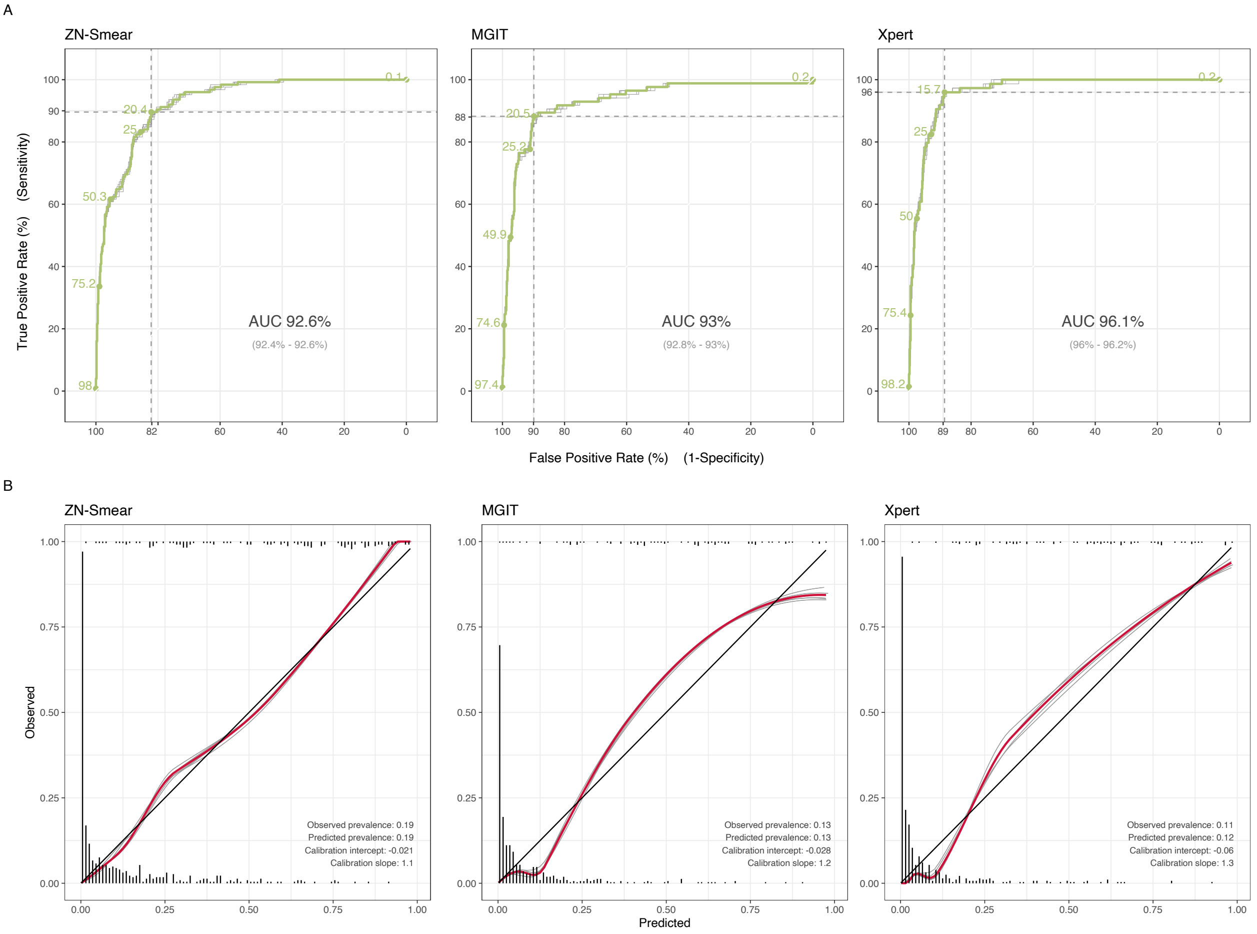

Figure S3: Performance of the selected indicator model. A: ROC and AUC against confirmatory test results. AUC values are presented as "average (min-max over 5 repetitions of cross-validation)"; B: Calibration plot, showing the relationship between the predicted probability and observed test results, smoothed by a loess curve. The grey lines are fitted curves from each of the five 20-fold cross-validations and coloured lines represent their average

S.2.4 Scaled Brier scores of the prevalence models against the final hospital diagnosis

This section compares the scaled Brier scores, also called explained variation or index of predictive accuracy (IPA), of the full and simplified prevalence models against the pseudo-gold standard final hospital diagnosis. In Formula:

$$
\begin{aligned}
B &= \frac{1}{N}\sum_{i=1}^{N}(\hat{y}_i - y_i)^2 \\
\mathbf{IPA} &= \frac{B_{\text{null}} - B}{B_{\text{null}}} = 1 - \frac{B}{B_{\text{null}}}
\end{aligned} \tag{10}
$$

where B is the Brier score, $y_i = 0, 1$ is the observed binary outcome, $0 \leq \hat{y}_i \leq 1$ is the predicted risk based on the prevalence model. B_{null} is the Brier score of the null model, i.e. the model that does not include any covariates. This means that everyone is given the same risk, which is the prevalence. The scaled Brier score **IPA** depicts how much predictive value is gained against the Null model. A scaled Brier score of 0 means that the performance of our model is identical to that of the Null model. The best value is 1 (our model has perfect prediction).

The scaled Brier score for the full model is 0.71 and for the simplified prevalence model, it is 0.50.

S.3 Assumption checks and sensitivity analysis

S.3.1 Methods

S.3.1.1 Latent class model

Independence of test results. Apart from model performance metrics, we also checked our conditional independence assumptions, both without (model 1) and with correction for latent bacillary burden (model 2-5). To do this, we plotted the residual pairwise correlation between confirmatory tests, effectively comparing predicted pairwise correlation and observed pairwise correlation [12].

Near-perfect specificity of GeneXpert. GeneXpert may not have near-perfect specificity [1]. Then some patients with missing Xpert that did not have TBM would have a positive test result. To check this, we did a sensitivity analysis in which we added the presence of an Xpert test as the fourth manifest variable to the indicator model. We assumed that observation of Xpert only depends on TBM status of the patient. It is denoted as *obs*.

For model 1, Formula (2) becomes

$$
\begin{aligned}
P_i &= P(y_i^{ZN\text{-}Smear} = s, y_i^{Mgit} = m, y_i^{Xpert} = x, obs_i = o) \\
&= \theta_i \times I(obs_i = 1) \times P(obs_i = 1|C_i = 1) \\
&\qquad \times P(y_i^{ZN\text{-}Smear} = s, y_i^{Mgit} = m, y_i^{Xpert} = x|C_i = 1, obs_i = 1) \\
&\quad + \theta_i \times I(obs_i = 0) \times (1 - P(obs_i = 1|C_i = 1)) \\
&\qquad \times P(y_i^{ZN\text{-}Smear} = s, y_i^{Mgit} = m|C_i = 1, obs_i = 0) \\
&\quad + (1 - \theta_i) \times I(obs_i = 1) \times P(obs_i = 1|C_i = 0) \\
&\qquad \times P(y_i^{ZN\text{-}Smear} = s, y_i^{Mgit} = m, y_i^{Xpert} = x|C_i = 0, obs_i = 1) \\
&\quad + (1 - \theta_i) \times I(obs_i = 0) \times (1 - P(obs_i = 1|C_i = 0)) \\
&\qquad \times P(y_i^{ZN\text{-}Smear} = s, y_i^{Mgit} = m|C_i = 0, obs_i = 0)
\end{aligned}
\tag{11}
$$

For the more extended models including bacillary burden, formulas translate analogously. There is a potential correlation between *obs* and test results as some factors can suggest the clinician to prescribe an Xpert test. For example, HIV infection increases the bacillary burden (and consequently the probability of having positive test results) and, at the same time but via a different pathway, prompts the clinician to perform the confirmatory tests. To correct for this, we regressed *obs* on some characteristics that may suggest TBM. In this analysis, we considered HIV, TB-suggestive symptoms and duration, and both Pulmonary TB and miliary TB on X-ray images and used the logistic regression model.

$$
\text{logit}[P(obs = 1|C = c)] = z_{obs}^c + V_{obs}^T \gamma_{obs}^c,
$$

where V_{obs} are the relevant characteristics that raise suspicion of TBM, γ_{obs} is the vector of coefficients, and z_{obs}^c is the intercept given TBM status $C = c \in \{0, 1\}$.

The priors for z_{obs}^c were chosen as follows. We used a weakly informative symmetric prior distribution around 0.5 for the non-TBM group and a moderately informative skewed prior distribution for the TBM group (Supplementary Figure S4) as we believe most patients in the TBM group have been tested with the

confirmatory tests. However, we widened the scale enough so that an extreme value of 50% TBM patients who left untested was allowed. Subsequently, we extended the scale of z^C_{obs} so that the 95% inter-percentile covered the extreme value of 0.1 (i.e, Xpert was only done for 10% of TBM group), this is denoted as the "Wide prior" model in Supplementary Figure S6.

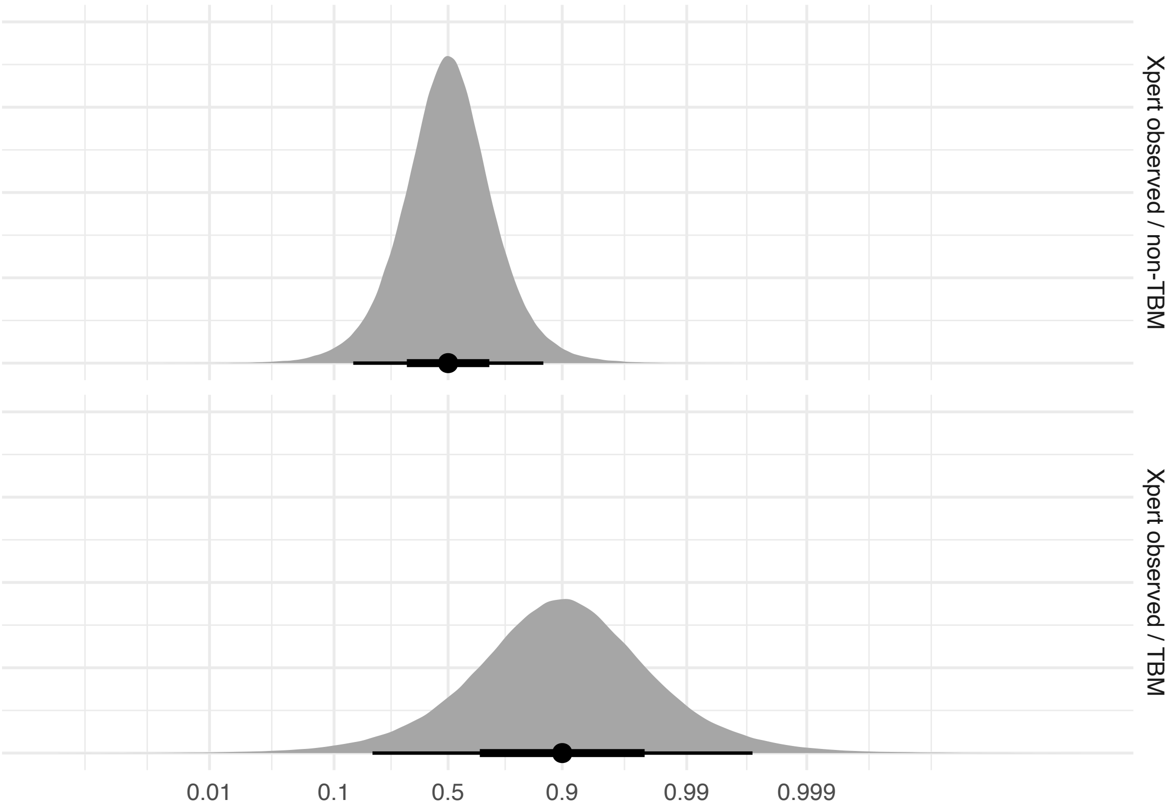

Figure S4: Prior distribution for Xpert response rate, in non-TBM (top) and TBM (bottom) patients without HIV. Point, thick lines, and thin lines are the median, 50% and 95% inter-percentile ranges. Logit scale is used along the x-axis

S.3.1.2 Imputation model

To assess the validity and impact of our imputation model, we used three different checks. First, we visually compared the distributions of the imputed data and observed data, conditional on the response propensity. To obtain the response propensity, for each variable of interest Y_v we fitted a logistic regression with the indicator of missingness as the response variable and the completed variables apart from Y_v (denoted as Y_{-v}) as the independent variables. We plotted Y_v against the propensity of response (e_v), using different colours for imputed and observed values of Y_v and added smoothing loess curves with a span of 0.8. Second, we compared the residual of $Y_v^{imputed}$ and $Y_v^{observed}$ conditionally on e_v [30]. To do this, we fitted a standard linear regression for continuous variables and a logistic regression for binary variables. The results were demonstrated using a kernel density plot for the former and loess curves for the other. Third, we performed a posterior predictive check in which we compared the posterior distribution of the coefficients of interest in the prevalence model based on the completed datasets (which contained observed and imputed values) and 100 replicated datasets in which all variables were imputed based on the imputation model [30,31]. Each replicated data set was obtained by selecting an iteration and using the values of the coefficients of the imputation models at that iteration to impute values for the variables with missing values for all individuals. This data set was used to obtain a posterior distribution of the coefficients in a separate MCMC procedure. For a good imputation, we would expect the distribution of the posteriors for the replicated data sets to be similar to the one for the completed data set.

For simplicity, we only analysed imputation results for variables that had more than 10 missing observations. As *TB-suggestive symptoms* and *focal neurological deficit* are expected to be MNAR on the composite level (Supplementary Table C1), we only performed the posterior predictive check for these two variables.

S.3.2 Results

S.3.2.1 Latent class model

Independence of test results. The expected residual correlations between 3 pairs of confirmatory tests for 5 model choices are shown in Supplementary Figure S5. Except for model 1 which underestimated the observed values, other models that incorporated random effects showed a good correspondence between predicted and observed correlation, in which case all residual values fluctuated around 0. This suggested that our correction for conditional dependence of manifest variables was indeed necessary.

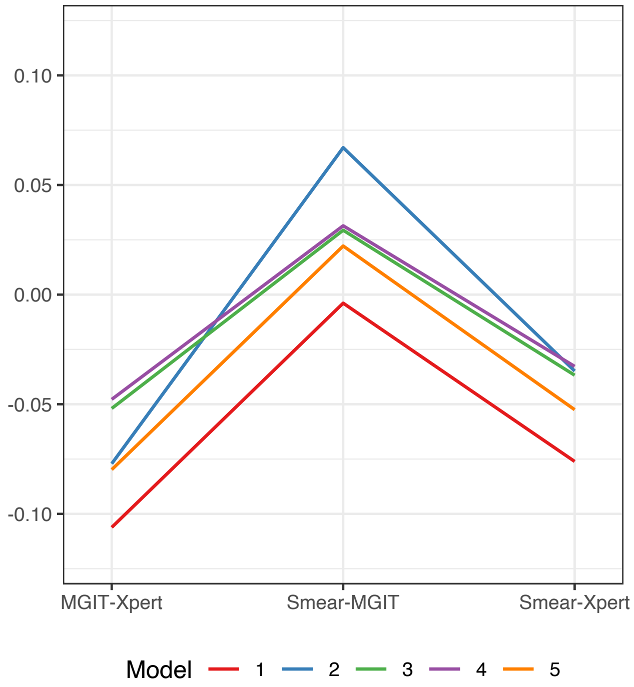

Figure S5: Residual correlation plots for five different model choices

Near-perfect specificity of GeneXpert. There were no large differences in the posterior estimates of the model with missing Xpert and the selected model (model 3) (Supplementary Figure S6), except for cranial nerve palsy and past noticed TB contact. Overall, the estimates of the selected model are shrunken more towards 0. The response probability for Xpert in the non-TBM group was estimated to be 0.31 (0.26 - 0.37). Almost all TBM patients were tested.

S.3.2.2 Imputation of covariables

No systematic differences were seen in imputed and observed values conditional on response propensity for HIV status, symptom duration, and RGCS (Supplementary Figure S7). In Supplementary Figure S8, for all coefficients and the intercept, both the posterior distribution and the estimated median based on the completed dataset do not fall out of the plausible counterparts based on the fully imputed datasets. This suggests that the difference in imputed and observed values does not heavily impact the posterior estimates. The strongest shift is for TB-suggestive symptoms, for which the estimate of the completed dataset is marginally lower than those of the replicated ones. Some discrepancies between completed and fully imputed datasets were expected, as TB-suggestive symptoms and Focal neurological deficit were MNAR on the composite level, as mentioned in the method part and in Supplementary Table C1. However, its median

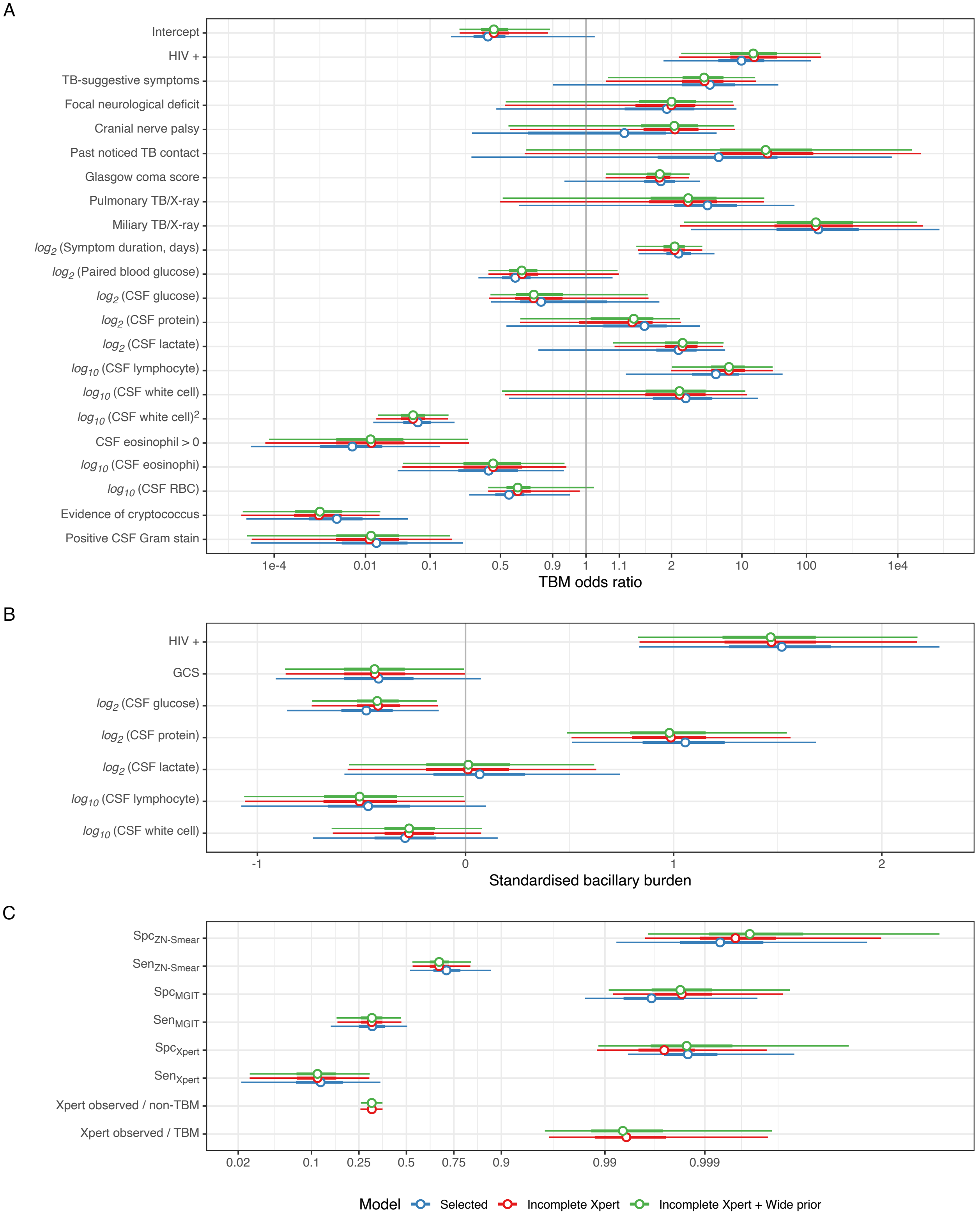

Figure S6: Estimates of model 3 (denoted as "Selected"), model 3 with missing Xpert (denoted as "Incomplete Xpert"), and model 3 with missing Xpert and a wider prior for test specificity (denoted as "Incomplete Xpert + Wide prior"). From top to bottom are estimates of the prevalence model, bacillary burden model, and sensitivity (TPR) and (1 - Specificity) (FPR) for 3 confirmatory tests and the response rates of Xpert for TBM and non-TBM patients without HIV. In all plots: dots, thick lines, and thin lines are the means, 50%, and 95% credible intervals.

is still within the 95% confidence interval and its posterior distribution still mostly overlaps the replicated datasets.

S.4 Analysis session info

- R version 4.2.1 (2022-06-23), `x86_64-pc-linux-gnu`
- Running under: `Ubuntu 18.04.6 LTS`
- Matrix products: default
- BLAS: `/usr/lib/x86_64-linux-gnu/blas/libblas.so.3.7.1`
- LAPACK: `/usr/lib/x86_64-linux-gnu/lapack/liblapack.so.3.7.1`
- Base packages: base, datasets, graphics, grDevices, methods, stats, utils
- Other packages: ggplot2 3.4.1
- Loaded via a namespace (and not attached): abind 1.4-5, assertthat 0.2.1, backports 1.4.1, bayesplot 1.10.0, bit 4.0.5, bit64 4.0.5, broom 1.0.3, callr 3.7.3, car 3.1-1, carData 3.0-5, caret 6.0-93, cellranger 1.1.0, checkmate 2.1.0, class 7.3-20, classifierplots 1.4.0, cli 3.6.1, cmdstanr 0.5.3, codetools 0.2-18, colorspace 2.1-0, compiler 4.2.1, coro 1.0.3, crayon 1.5.2, curl 5.0.0, data.table 1.14.8, DBI 1.1.3, digest 0.6.31, distributional 0.3.1, dplyr 1.1.0, fansi 1.0.4, farver 2.1.1, foreach 1.5.2, future 1.31.0, future.apply 1.10.0, generics 0.1.3, gghighlight 0.4.0, ggpubr 0.6.0, ggsignif 0.6.4, ggtext 0.1.2, globals 0.16.2, glue 1.6.2, gower 1.0.1, grid 4.2.1, gridExtra 2.3, gridtext 0.1.5, gtable 0.3.1, hardhat 1.2.0, import 1.3.0, inline 0.3.19, ipred 0.9-13, iterators 1.0.14, jsonlite 1.8.4, knitr 1.42, labeling 0.4.2, LaplacesDemon 16.1.6, lattice 0.20-45, lava 1.7.2.1, lifecycle 1.0.3, listenv 0.9.0, loo 2.5.1, lubridate 1.9.2, magrittr 2.0.3, MASS 7.3-58, Matrix 1.5-3, matrixStats 0.63.0, ModelMetrics 1.2.2.2, munsell 0.5.0, nlme 3.1-157, nnet 7.3-17, pander 0.6.5, parallel 4.2.1, parallelly 1.34.0, pbmcapply 1.5.1, pillar 1.8.1, pkgbuild 1.4.0, pkgconfig 2.0.3, plyr 1.8.8, png 0.1-8, posterior 1.4.1, prettyunits 1.1.1, pROC 1.18.0, processx 3.8.0, prodlim 2019.11.13, progressr 0.13.0, ps 1.7.2, purrr 1.0.1, R6 2.5.1, RColorBrewer 1.1-3, Rcpp 1.0.10, RcppParallel 5.1.7, readxl 1.4.2, recipes 1.0.5, reshape 0.8.9, reshape2 1.4.4, rlang 1.0.6, rmda 1.6, ROCR 1.0-11, rpart 4.1.16, rstan 2.26.15, rstatix 0.7.2, rstudioapi 0.14, scales 1.2.1, sessioninfo 1.2.2, splines 4.2.1, StanHeaders 2.26.15, stats4 4.2.1, stringi 1.7.12, stringr 1.5.0, survival 3.2-12, td.misc 0.0.0.9001, tensorA 0.36.2, tibble 3.1.8, tidyr 1.3.0, tidyselect 1.2.0, timechange 0.2.0, timeDate 4022.108, tools 4.2.1, torch 0.9.1.9001, utf8 1.2.3, V8 4.2.2, vctrs 0.5.2, withr 2.5.0, xfun 0.37, xml2 1.3.3

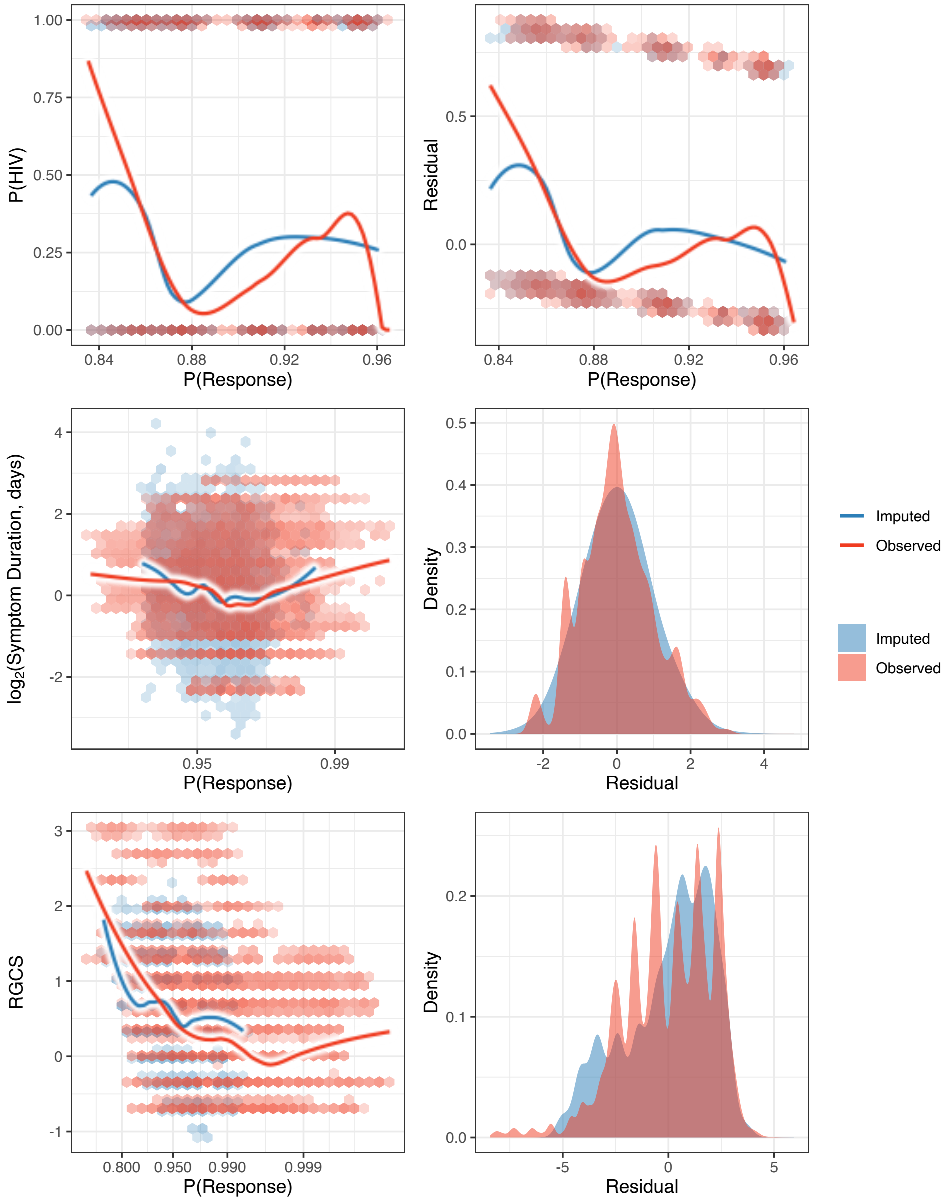

Figure S7: Diagnostic plots for the imputation model. On the left are hexagonal heatmaps between the response propensity and the values observed or imputed, centred and standardised. On the right-hand side are the kernel density plots of the residuals for continuous variables and the smoothed curve of the residuals against the response propensity for the binary variable HIV status, for the regression model $y \sim e_y$ where y is the variable of interest and e_y is the response propensity of that variable.

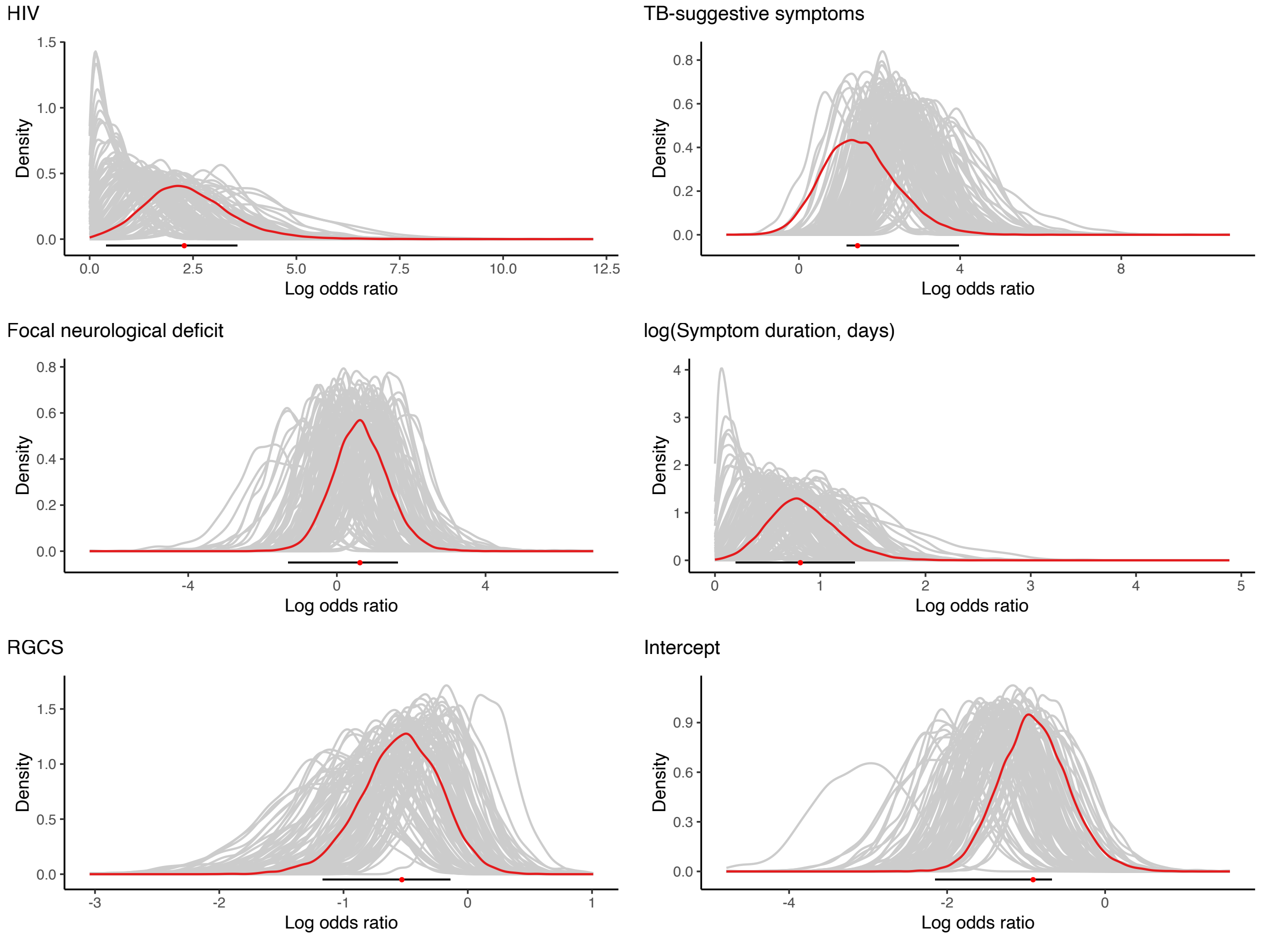

Figure S8: Posterior distributions of parameters for variables with missing values in the prevalence model. The curves are the posterior distributions based on the completed dataset in red and based on 100 replicated datasets in grey. The red point is the posterior median based on the completed dataset. The black lines are the 95% confidence intervals of the posterior medians of the replicated datasets.
